# Supplementary figures and images for: Viral proteogenomic and expression profiling during productive replication of a skin-tropic herpesvirus in the natural host
Source: PLoS Pathog. 2023 Jun 8;19(6):e1011204. doi: 10.1371/journal.ppat.1011204 (PMC10284419; doi:10.1371/journal.ppat.1011204)

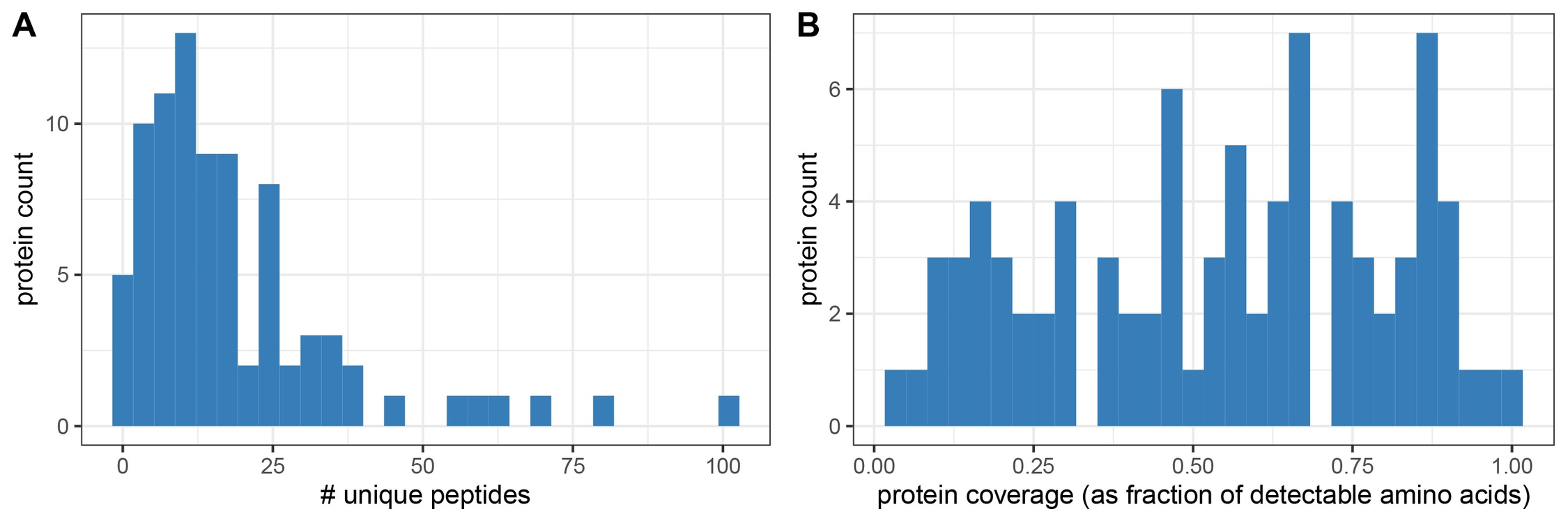

Supplement: S1 Fig — (A) Frequency of proteins by the number of unique peptides identified from them. (B) Frequency of proteins by their amino acid coverage, defined as the proportion of amino acids in the sequence likely to be detected by MS/MS (tryptic peptides ≥ 6 aa) that are covered by detected peptides. (TIF) [file ppat.1011204.s001.tif]

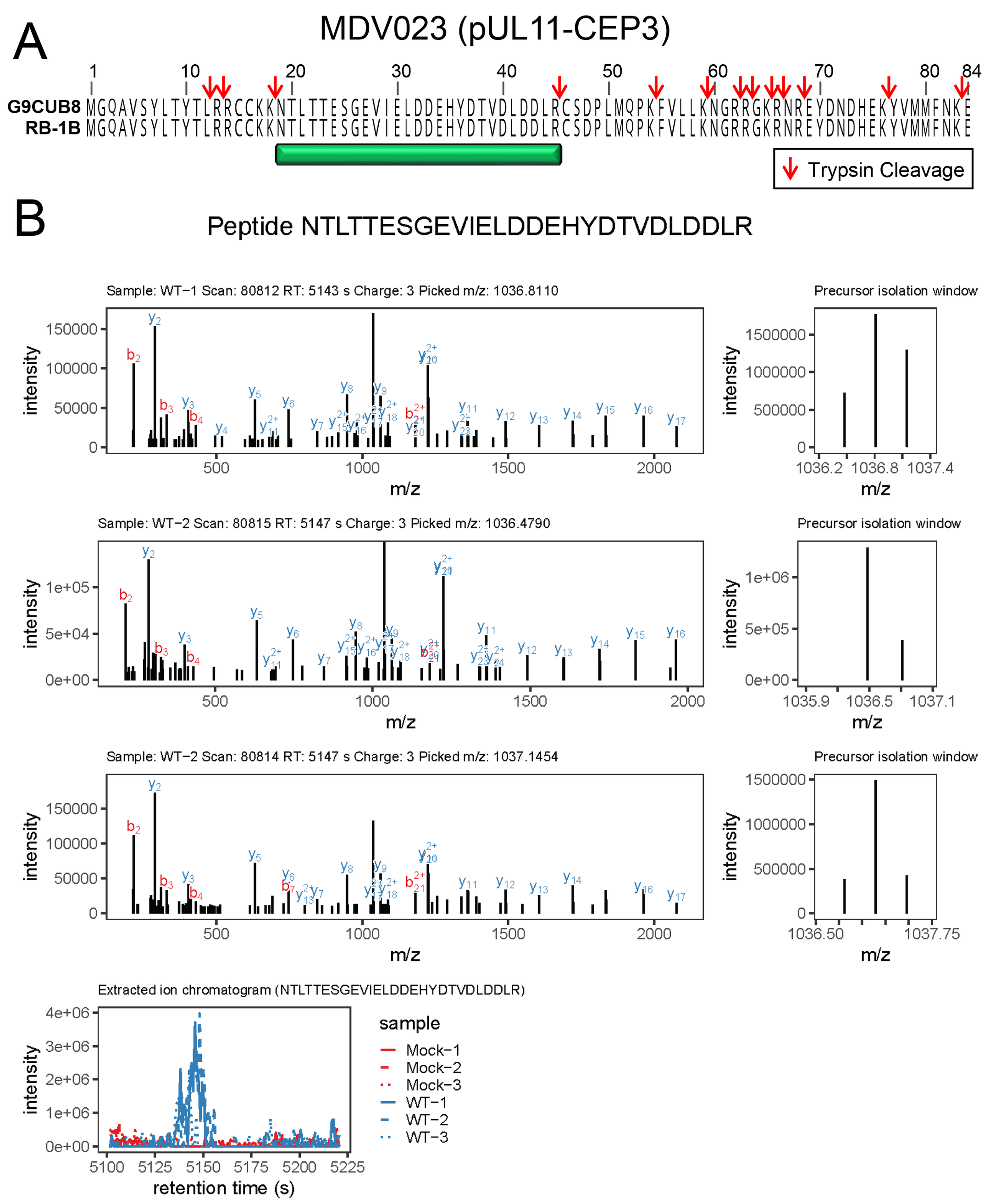

Supplement: S2 Fig — (A) Protein sequence of MDV023 (pUL11) comparing the reference sequence (G9CUB8) and the RB-1B strain used in this study, plus the predicted tryptic cleavage sites. (B) Representative MS2 spectra with annotated b/y ion series, and XIC elution profiles of the peptide mass in replicates of mock- and infected samples. (TIF) [file ppat.1011204.s002.tif]

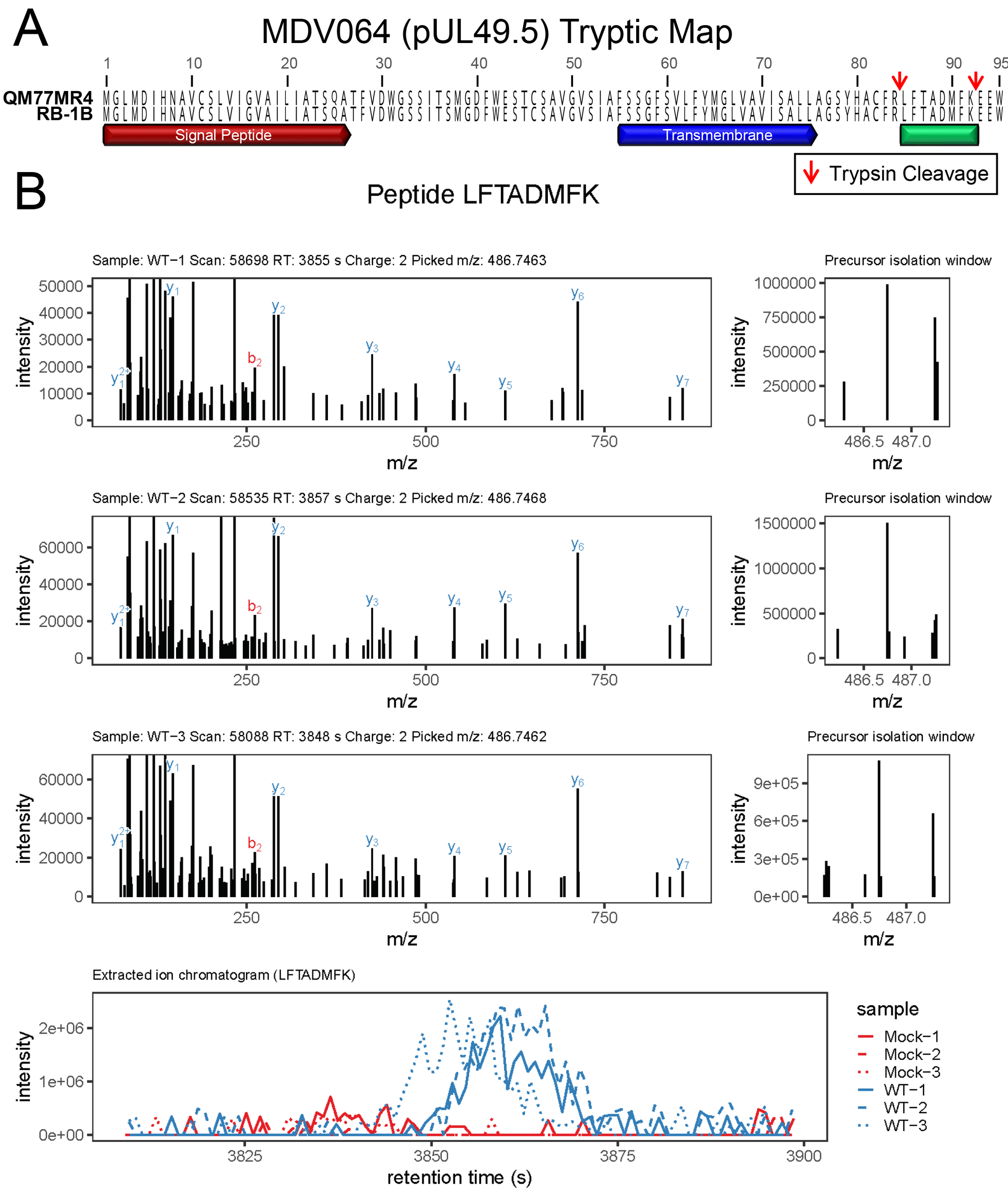

Supplement: S3 Fig — (A) Protein sequence of MDV064 (pUL49.5-gN) comparing the reference sequence (QM77MR4) and the RB-1B strain used in this study, plus the predicted tryptic cleavage sites, predicted signal peptide and transmembrane regions. (B) Representative MS2 spectra with annotated b/y ion series, and XIC elution profiles of the peptide mass in replicates of mock- and infected samples. (TIF) [file ppat.1011204.s003.tif]

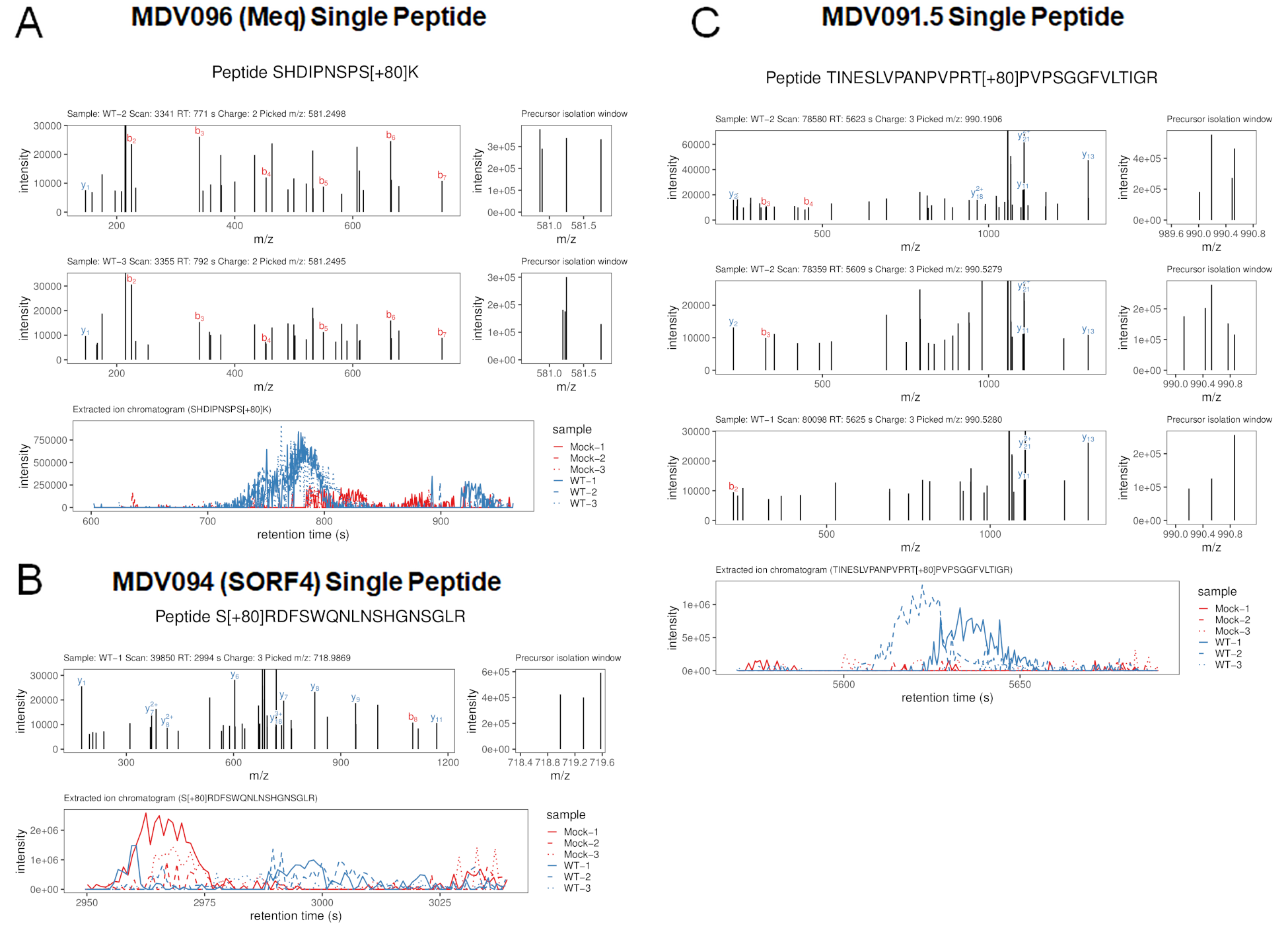

Supplement: S4 Fig — Representative MS2 spectra with annotated b/y ion series, and XIC elution profiles of the peptide mass in replicates of mock- and infected samples for MDV096 (A), MDV094 (B), and MDV0915 (C), indicating poor support for their correct identifications. (TIF) [file ppat.1011204.s004.tif]

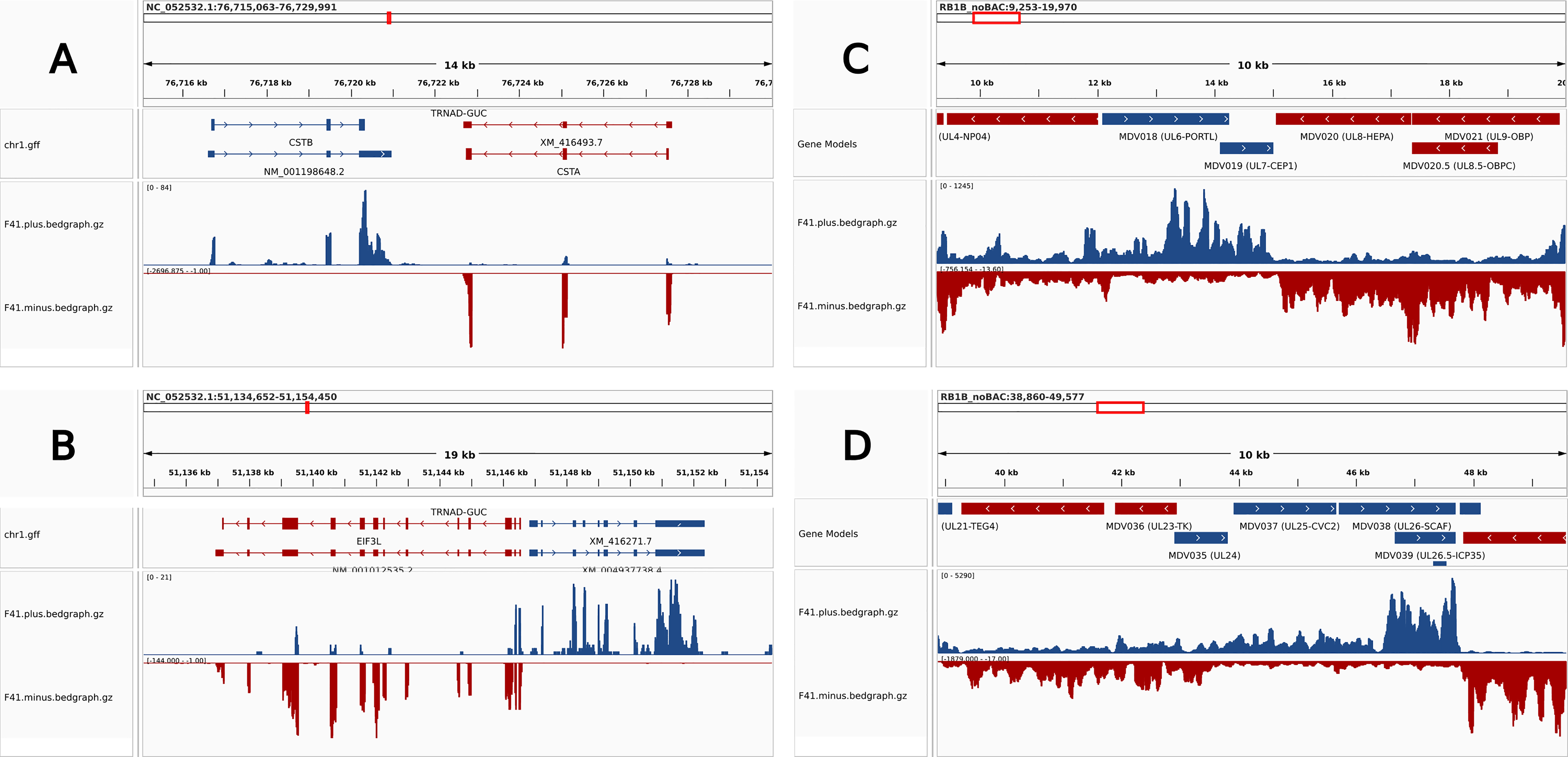

Supplement: S5 Fig — (TIF) [file ppat.1011204.s005.tif]

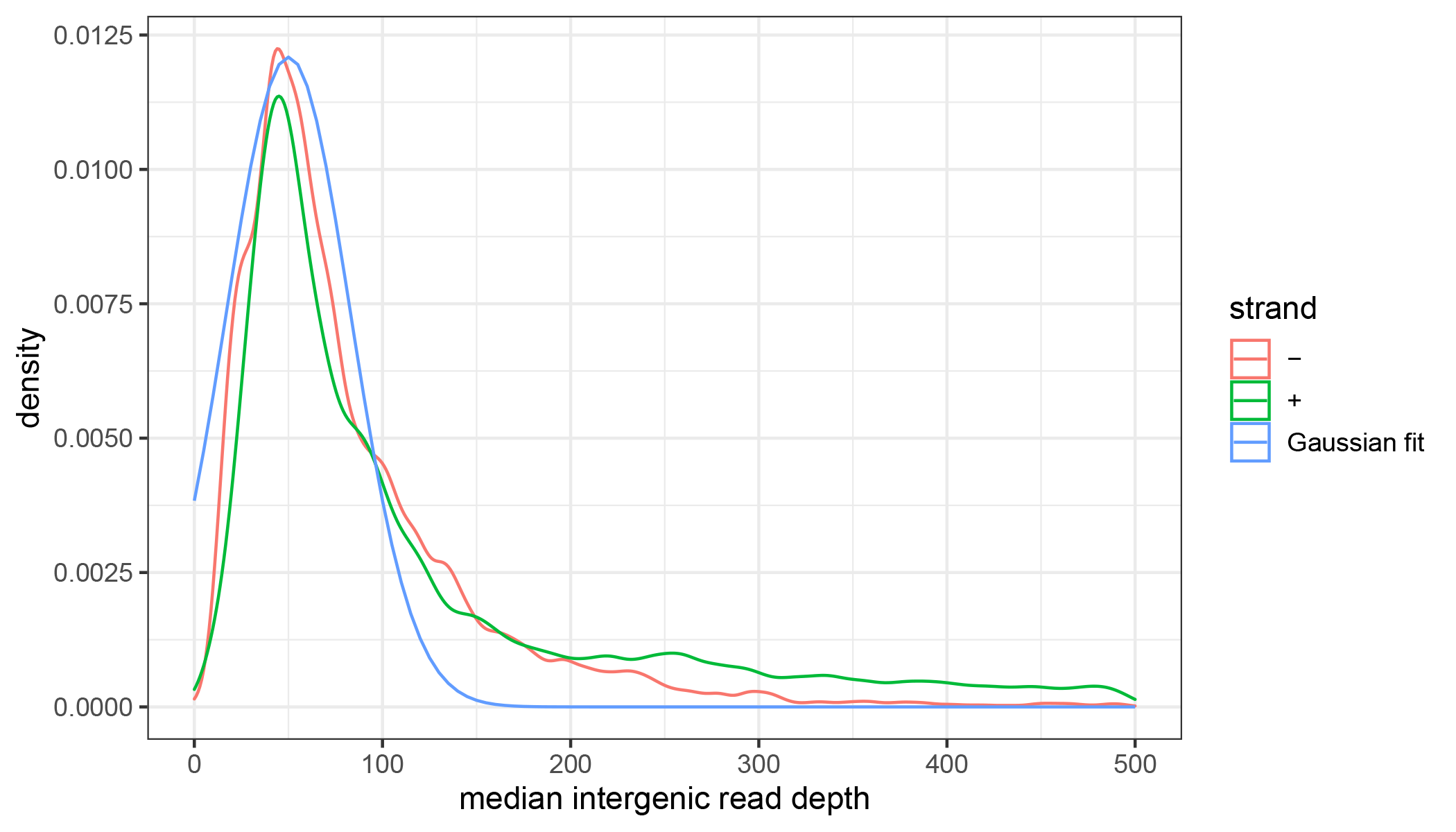

Supplement: S6 Fig — Shown are kernel density estimates for read coverage on forward (green) and reverse (red) strands calculated for each genomic position in assumed intergenic and antisense regions. A Gaussian distribution was fit to the data with mean = 50 and SD = 33 (blue). These values were used as estimates of background read coverage to calculate the threshold for true expression (mean + 2 SD). (TIF) [file ppat.1011204.s006.tif]

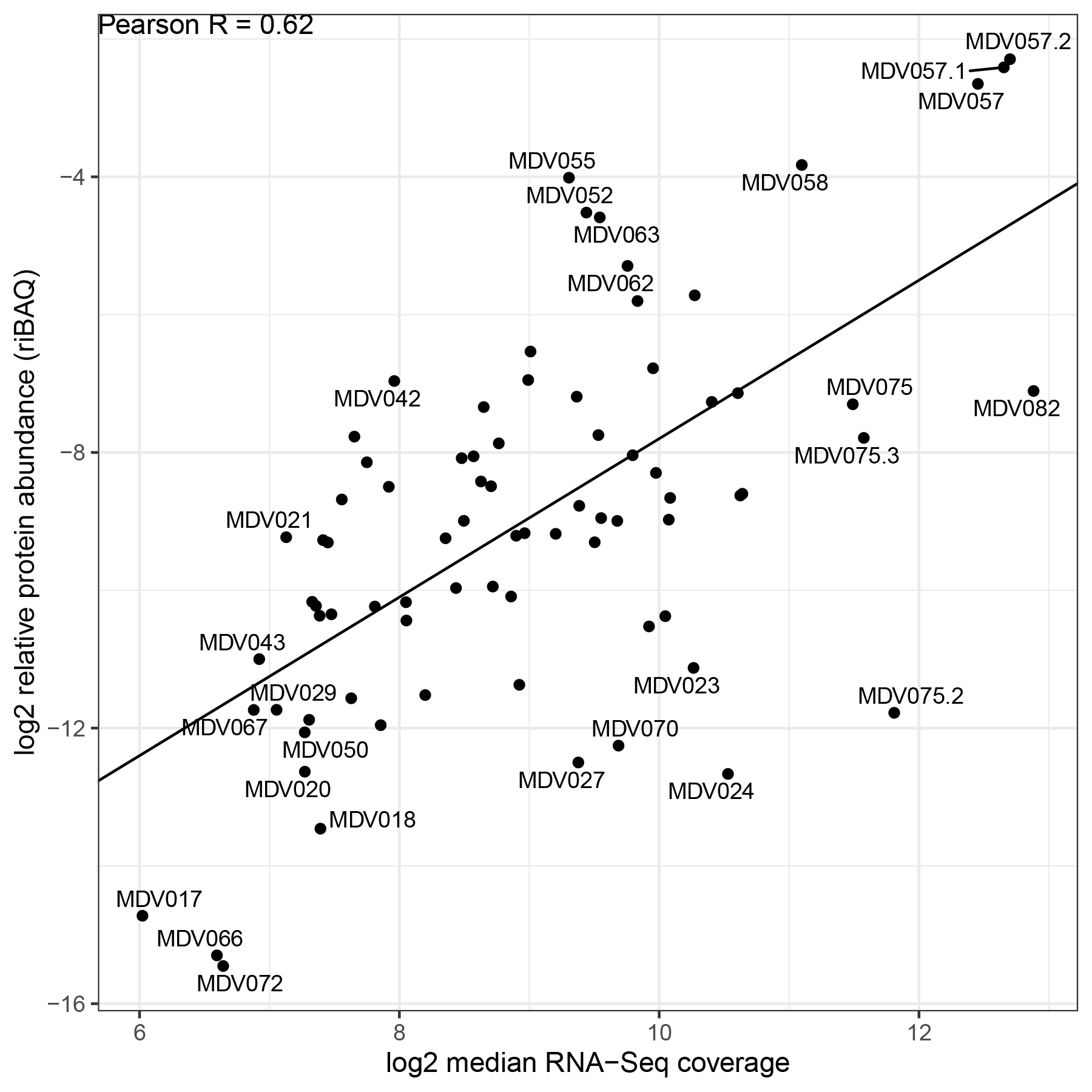

Supplement: S7 Fig — Shown is a scatterplot of log2-transformed median short-read RNA-Seq read depth and log2 relative protein abundance (from riBAQ calculation) for each protein detected by both methods. Outliers are labeled with gene identifiers. (TIF) [file ppat.1011204.s007.tif]

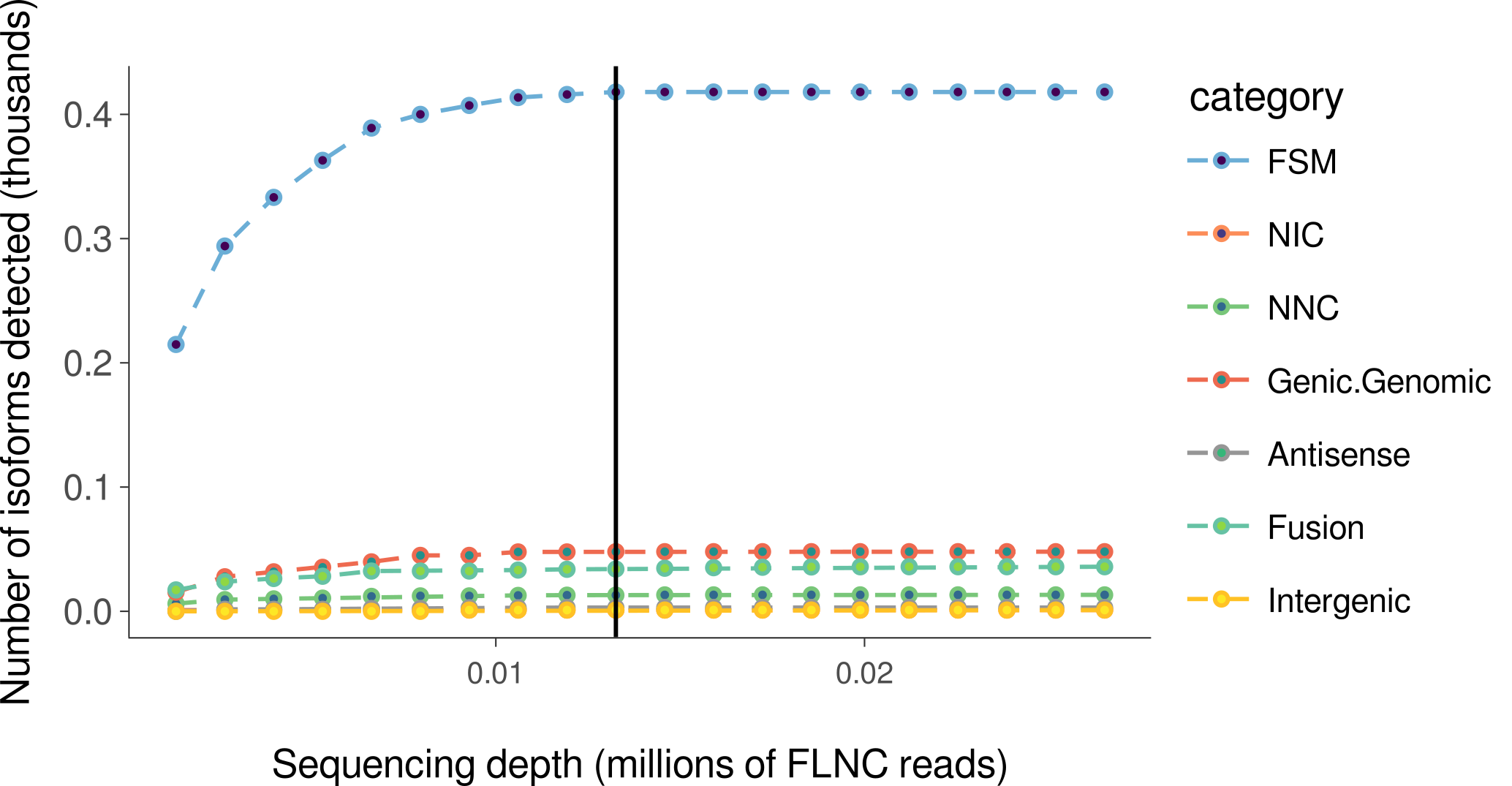

Supplement: S8 Fig — Shown is the saturation curve from SQANTI3 rarefaction analysis, with isoform count as a function of read depth. The vertical black line shows the actual sequencing depth of the experiment, and the points to the right of this line are extrapolated. FSM = full splice match; NIC = novel in catalog; NNC = novel not in catalog. (TIF) [file ppat.1011204.s008.tif]

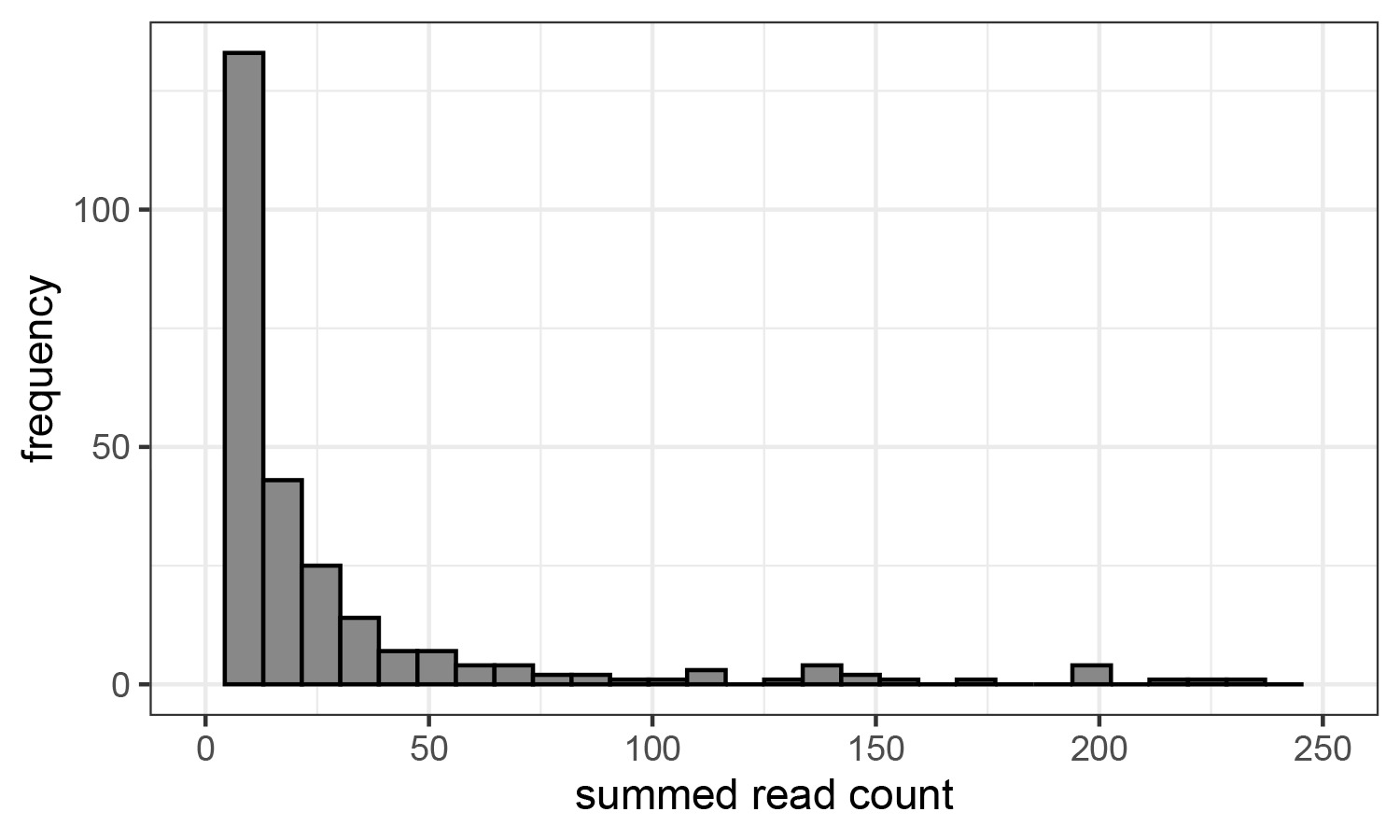

Supplement: S9 Fig — Counts represent the sum of FLNC reads assigned to each transcript by the isoseq3 software. (TIF) [file ppat.1011204.s009.tif]

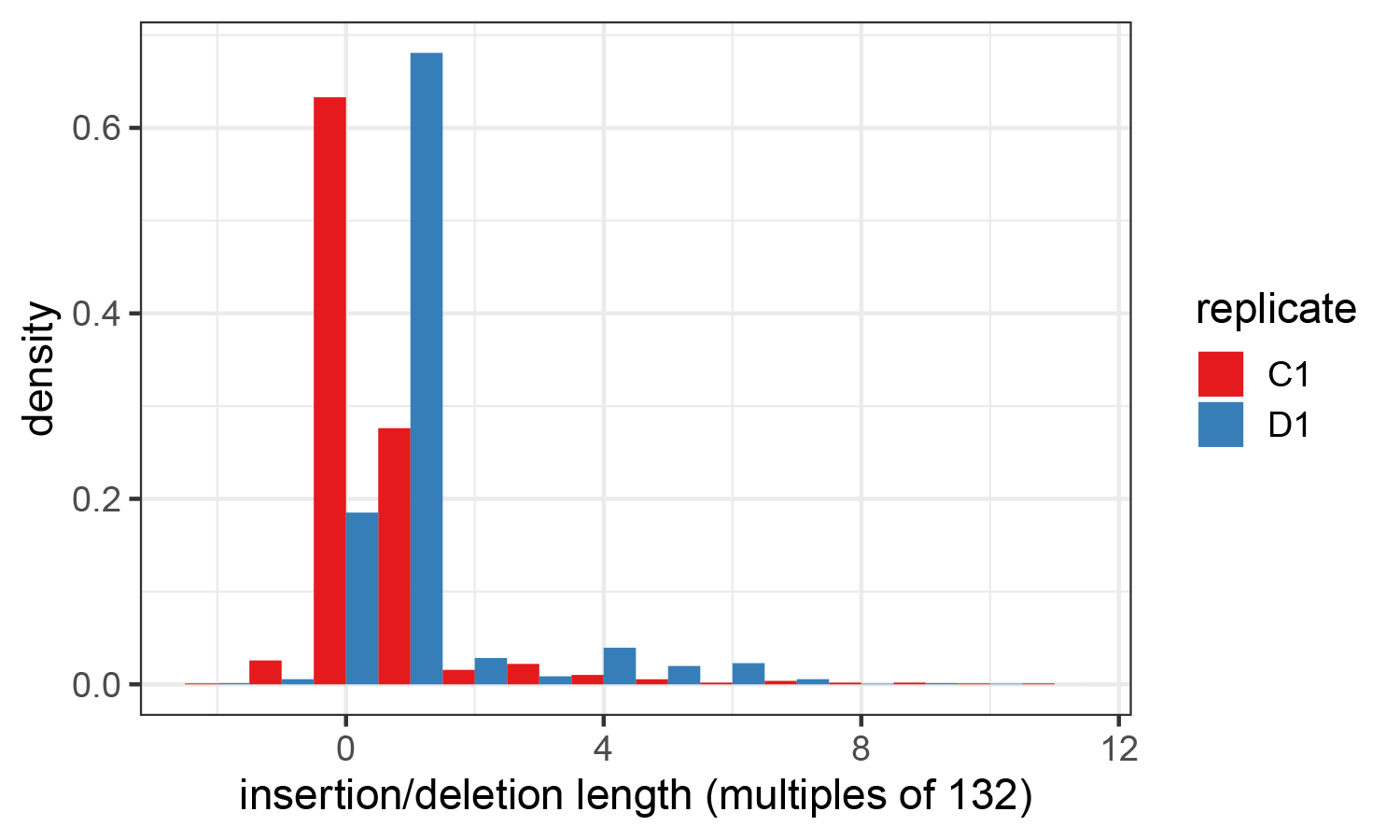

Supplement: S10 Fig — Long reads overlapping the 132 bp repeat region were extracted, and the repeat copy number for each was tabulated. Copy numbers in the plot are expressed relative to the three copies present in the reference genome (e.g., +1 represents four copies, -1 represents two copies). (TIF) [file ppat.1011204.s010.tif]

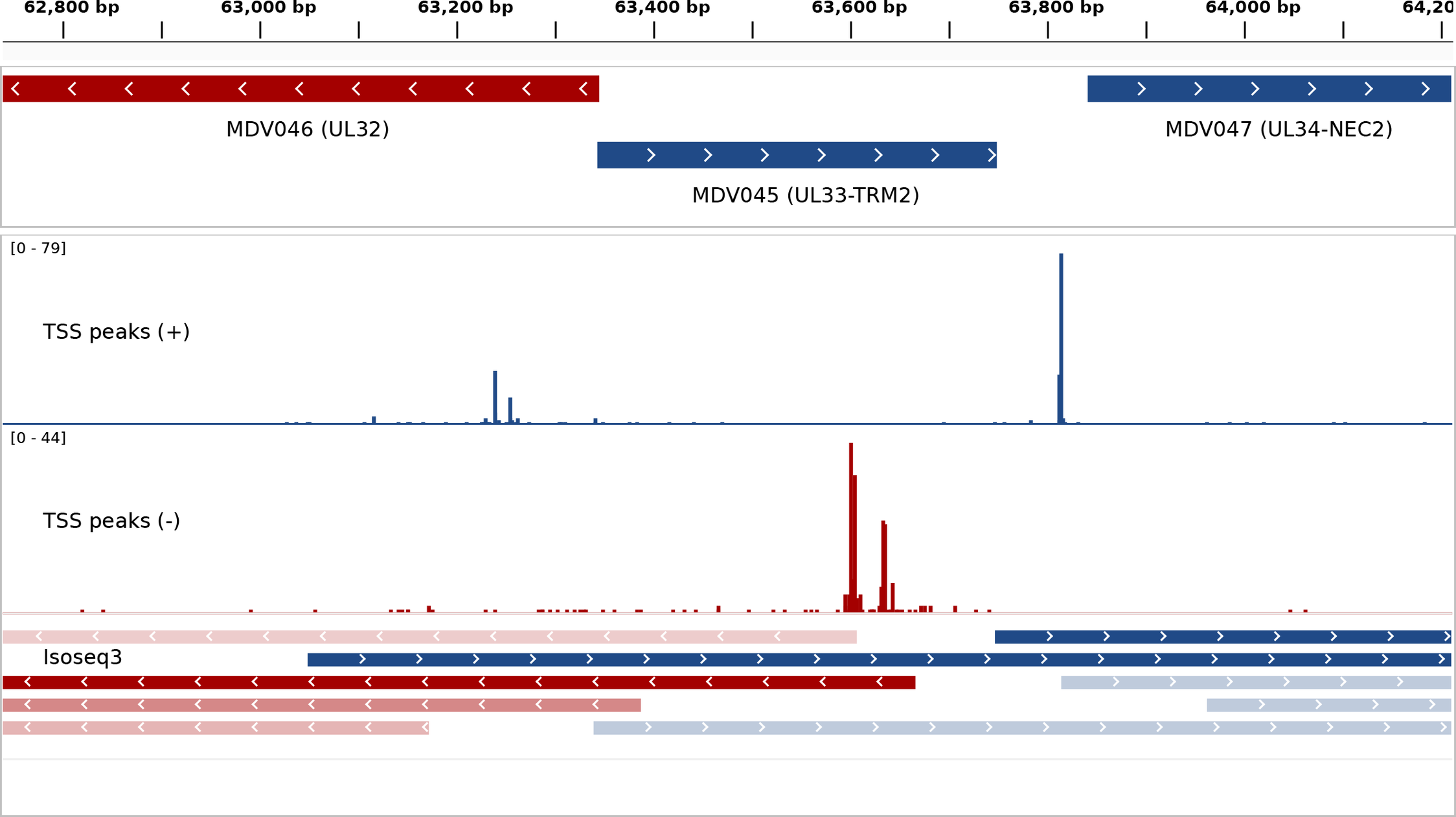

Supplement: S11 Fig — Shown is a representative region of the MDV genome in the IGV browser, with TSS peak tracks (+/- strands) as well as the annotated coding sequence and predicted transcript models from isoseq3, demonstrating the relative inaccuracy of the isoseq3 transcript 5’ ends under the settings used as compared with actual read start coverage (TSS peaks) at each position. (TIF) [file ppat.1011204.s011.tif]

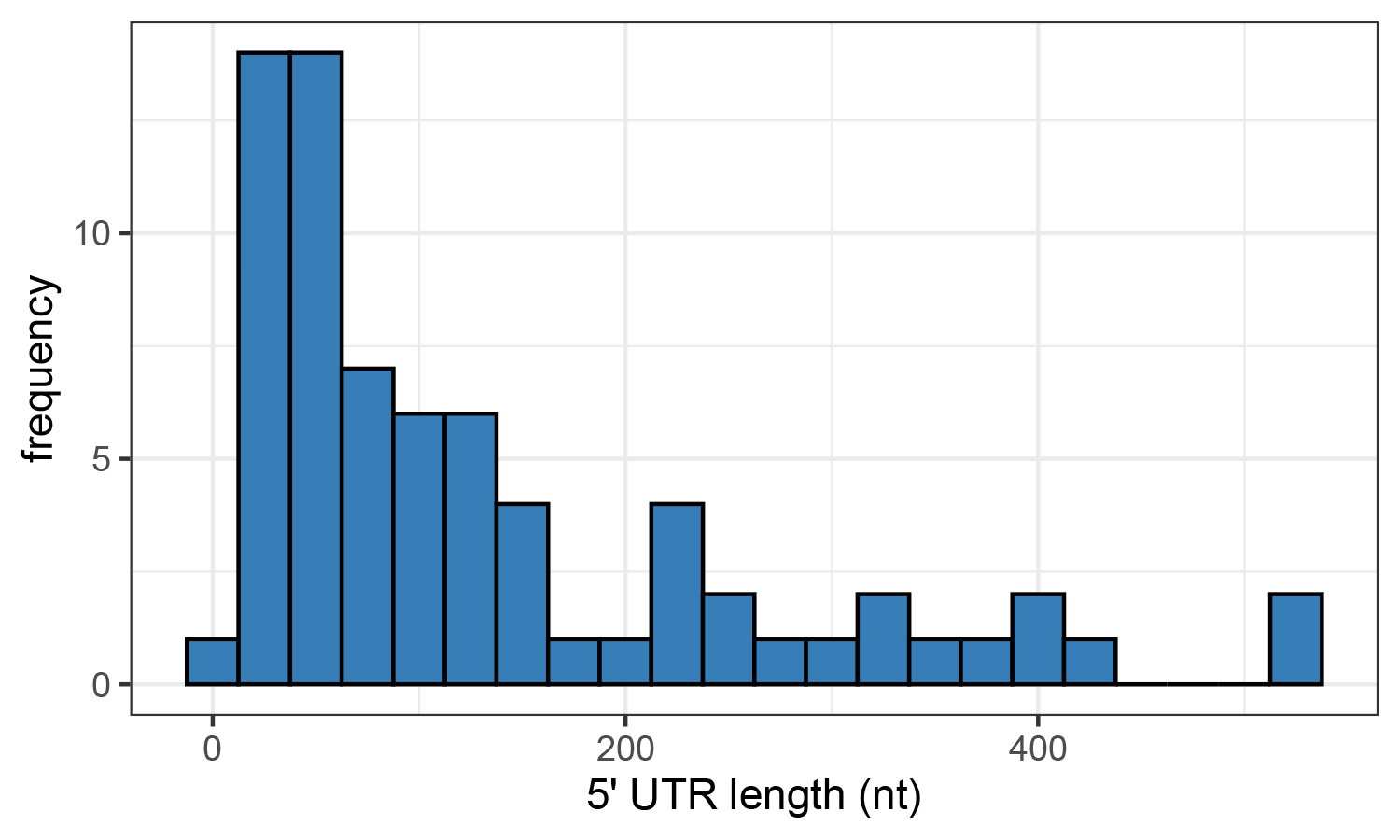

Supplement: S12 Fig — 5’UTRs were determined for all genes assigned primary TSS, and the length calculation accounted for introns within the 5’ UTRs of MDV008, MDV018, MDV037. (TIF) [file ppat.1011204.s012.tif]

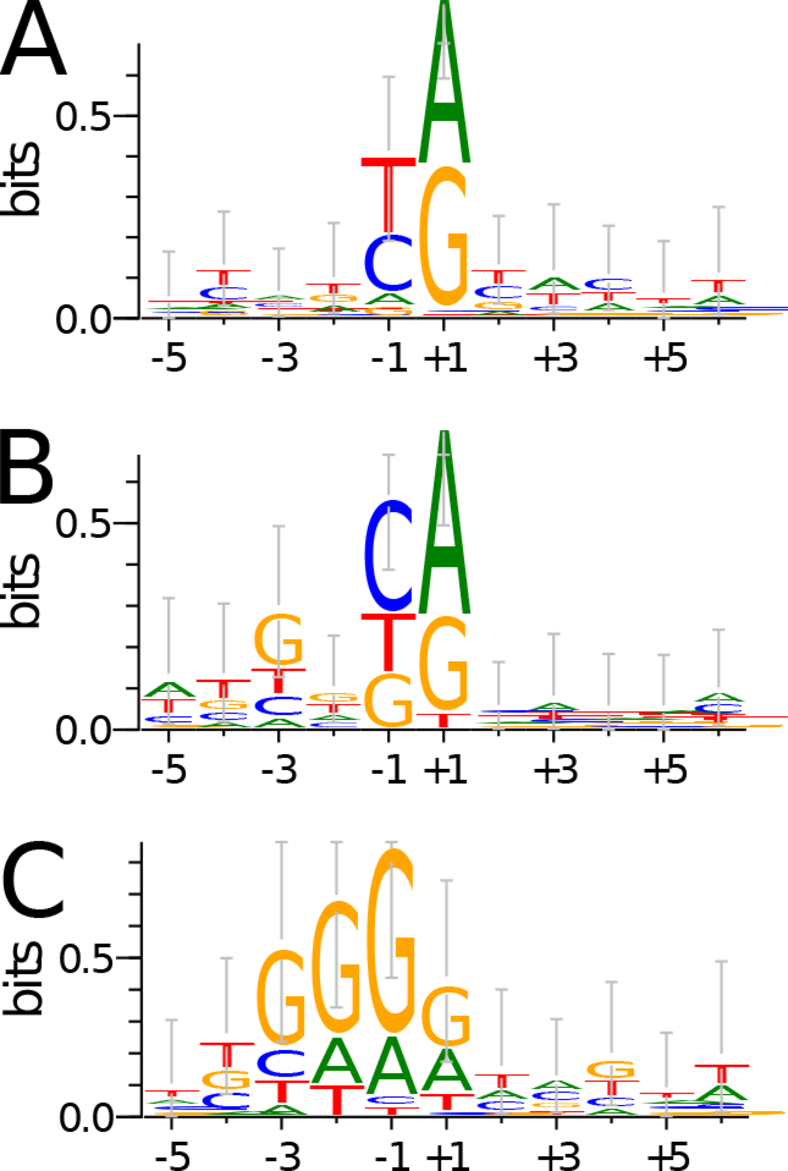

Supplement: S13 Fig — Shown are the sequence logos for the genomic sequence ± 5 bp of the (A) major TSS (as determined from IsoSeq read alignments), (B) secondary TSS, and (C) major + secondary TSS which do not contain a pyrimidine-purine dinucleotide at the TSS. (TIF) [file ppat.1011204.s013.tif]

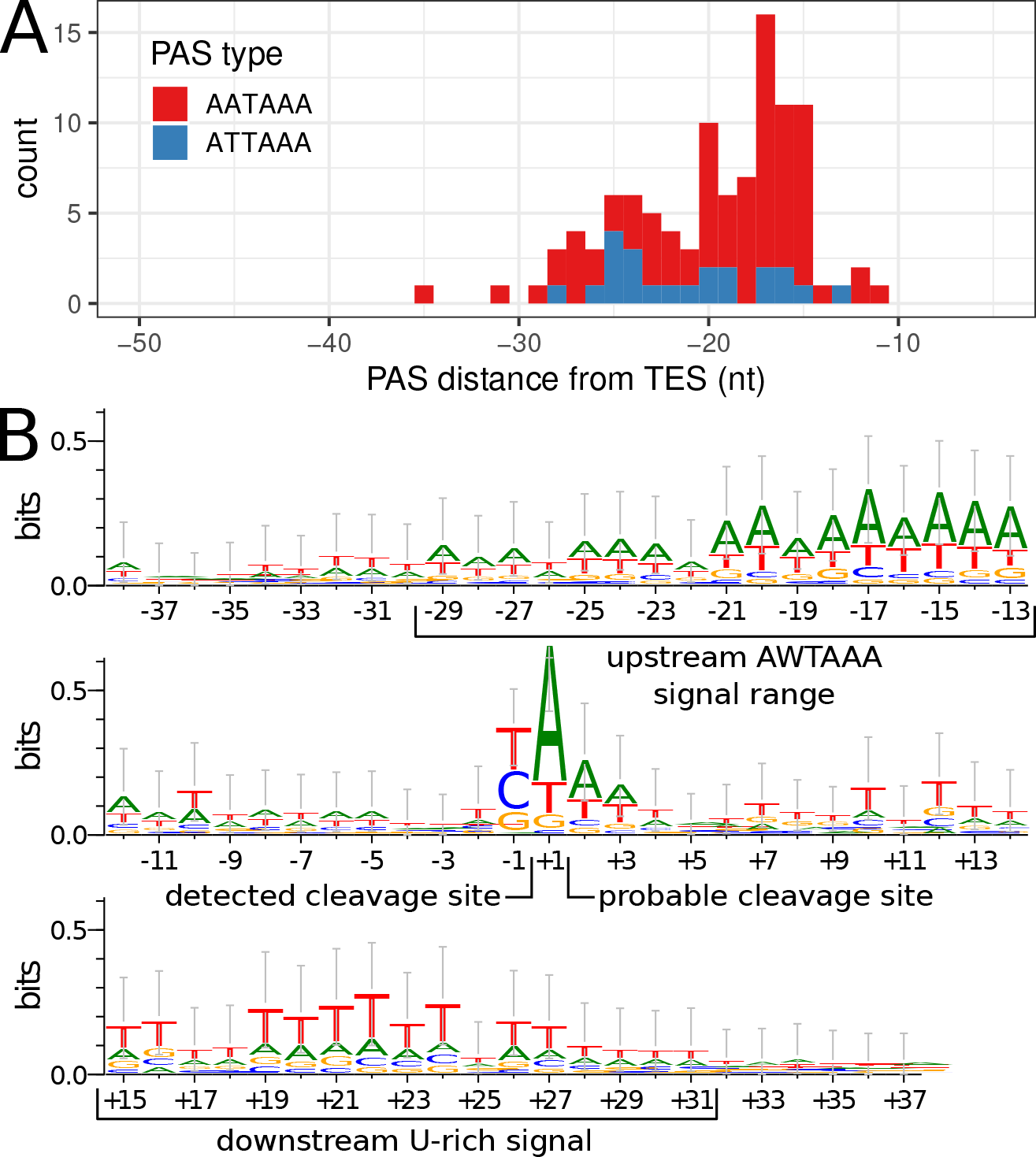

Supplement: S14 Fig — (A) Distance from the site of cleavage/polyadenylation (as determined by IsoSeq) to the upstream polyadenylation signal hexamer. (B) Sequence conservation logo for the surrounding genomic context centered on the cleavage/polyadenylation site. The plot is numbered from the cleavage location as determined by FLNC read termination, but the observed site is likely to be 1 bp upstream of the actual site as a result of the terminal adenine being removed by the IsoSeq processing software during poly-A tail removal. The upstream region of the conserved hexamer motif and downstream U(T)-rich region are clearly visible. (TIF) [file ppat.1011204.s014.tif]

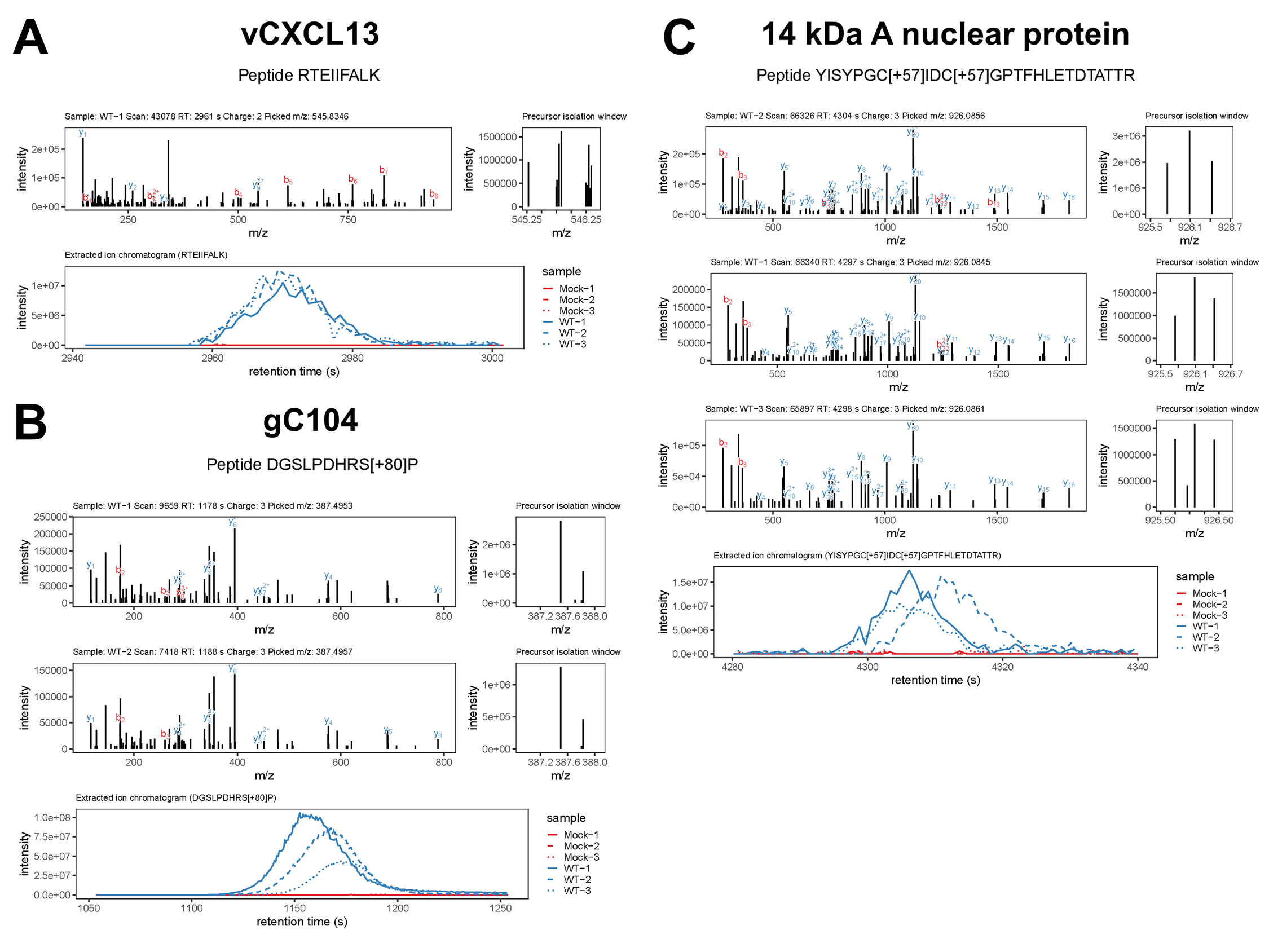

Supplement: S15 Fig — Shown for each peptide are MS2 spectra for the top PSMs annotated with b/y ion series, as well as aligned XIC elution profiles for the peptide mass for each of the six replicates. Each peptide is found only in infected replicates, as indicated by the XIC plots. (TIF) [file ppat.1011204.s015.tif]

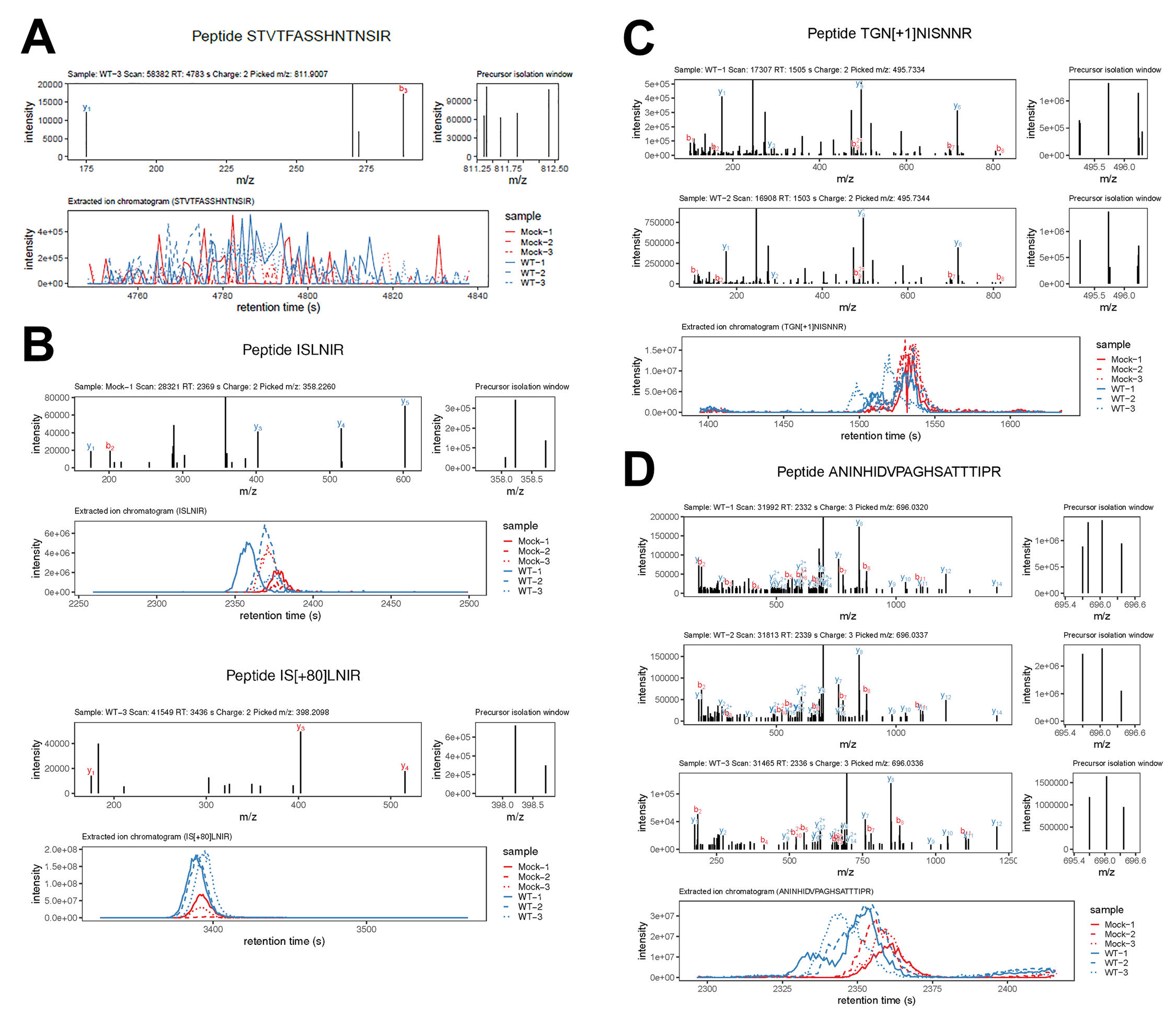

Supplement: S16 Fig — Shown for each peptide are MS2 spectra for the top PSMs annotated with b/y ion series, as well as aligned XIC elution profiles for the peptide mass for each of the six replicates. Each peptide is found only in infected replicates, as indicated by the XIC plots. These peptide identifications are poorly supported due to a combination of weak ion series and/or XICs showing the precursor mass eluting in both infected and mock replicates. (TIF) [file ppat.1011204.s016.tif]

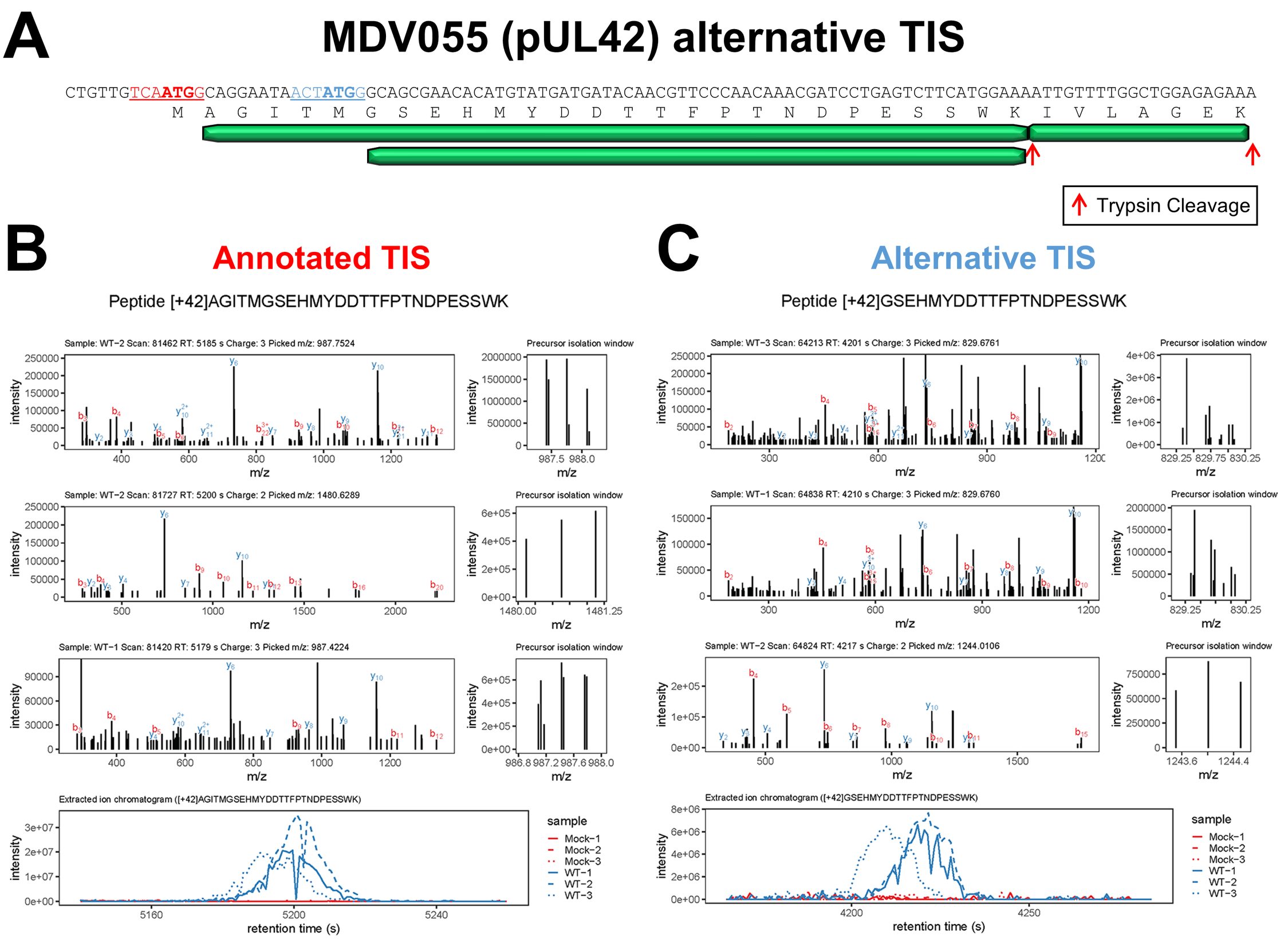

Supplement: S17 Fig — (A) 5’ end of MDV055 showing N-terminal peptides for both the annotated and alternative TIS identified by peptides. (B & C) For each peptide, annotated MS2 spectra for the top PSMs and six-replicate aligned XICs are shown. (TIF) [file ppat.1011204.s017.tif]

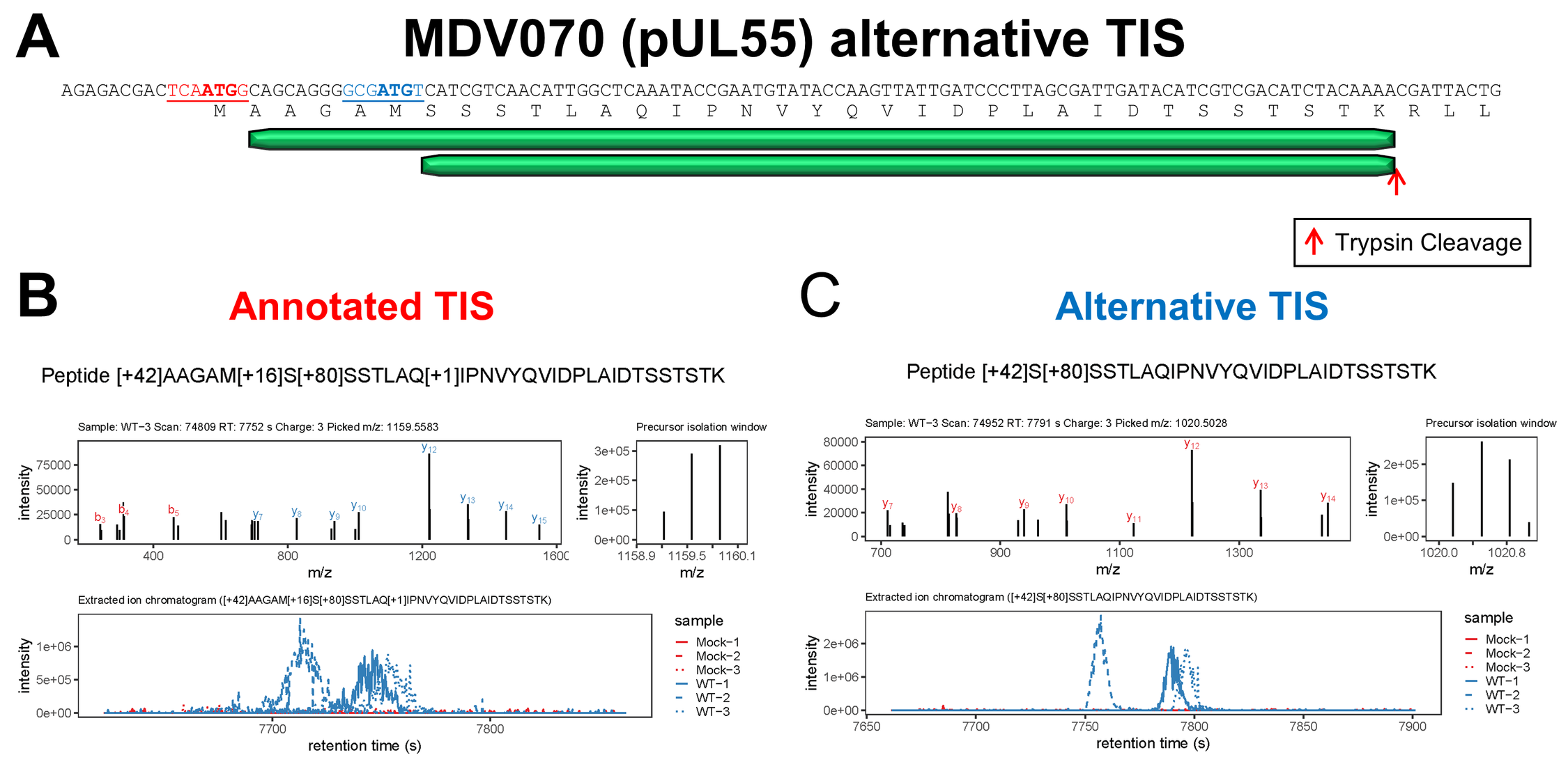

Supplement: S18 Fig — (A) 5’ end of MDV070 showing N-terminal peptides for both the annotated and alternative TIS identified by peptides. (B & C) For each peptide, annotated MS2 spectra for the top PSMs and six-replicate aligned XICs are shown. (TIF) [file ppat.1011204.s018.tif]

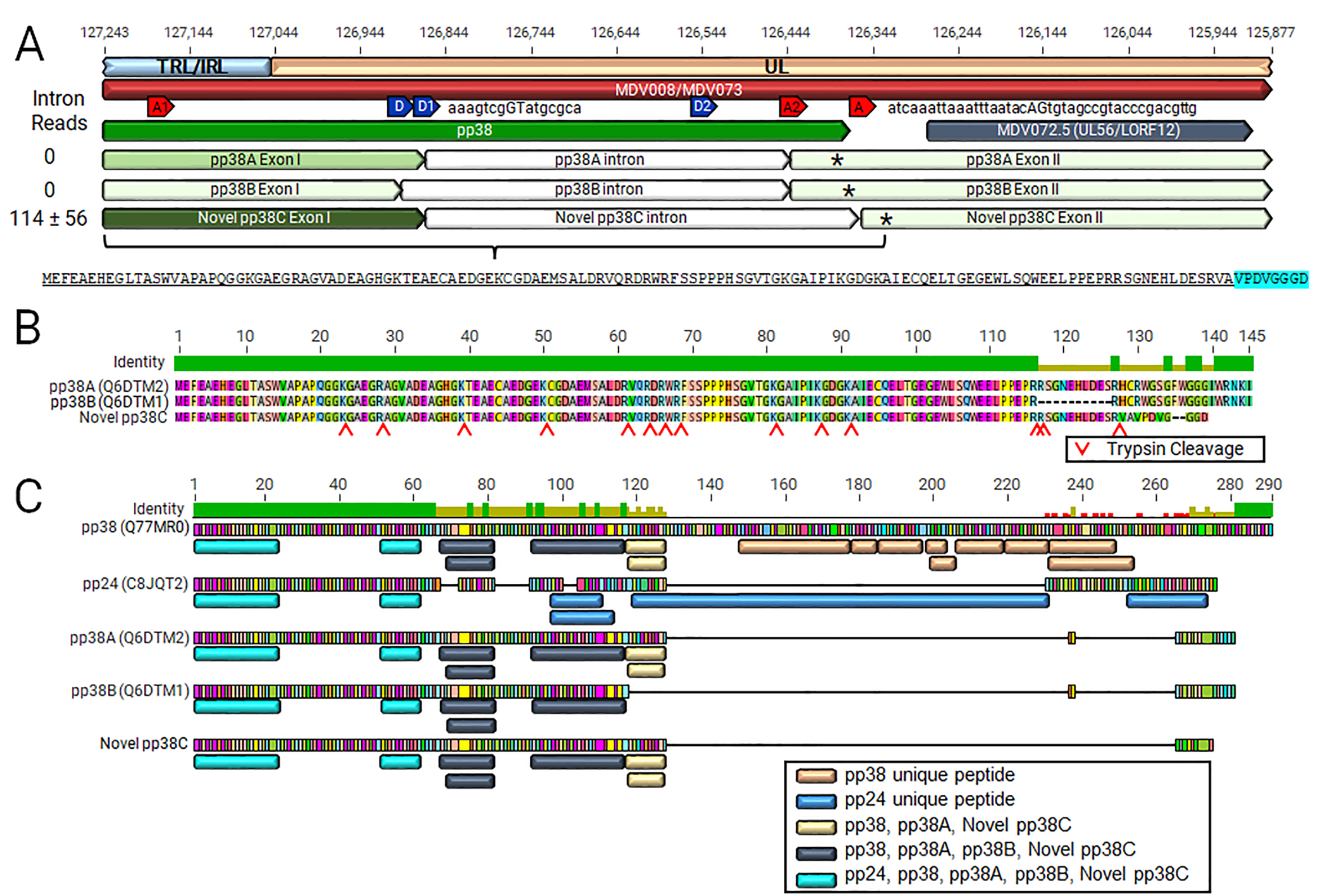

Supplement: S19 Fig — (A) MDV008 and MDV073 overlap the junction between the UL and RL regions, creating alternative proteins including previously identified pp38 and pp24, and pp38A and pp38B created through alternative splicing. A novel splice variant termed Novel pp38C is expressed in epithelial skin cells. Donor (“D”) and acceptor (“A”) locations are shown. (B) MUSCLE alignment of pp38A, pp38B, and Novel pp38C with trypsin cleavage sites. (C) MUSCLE alignment of pp38, pp24, pp38A, pp38B, and Novel pp38C and peptides detected in epithelial skin cells. Some peptides are unique to specific proteins. (TIF) [file ppat.1011204.s019.tif]

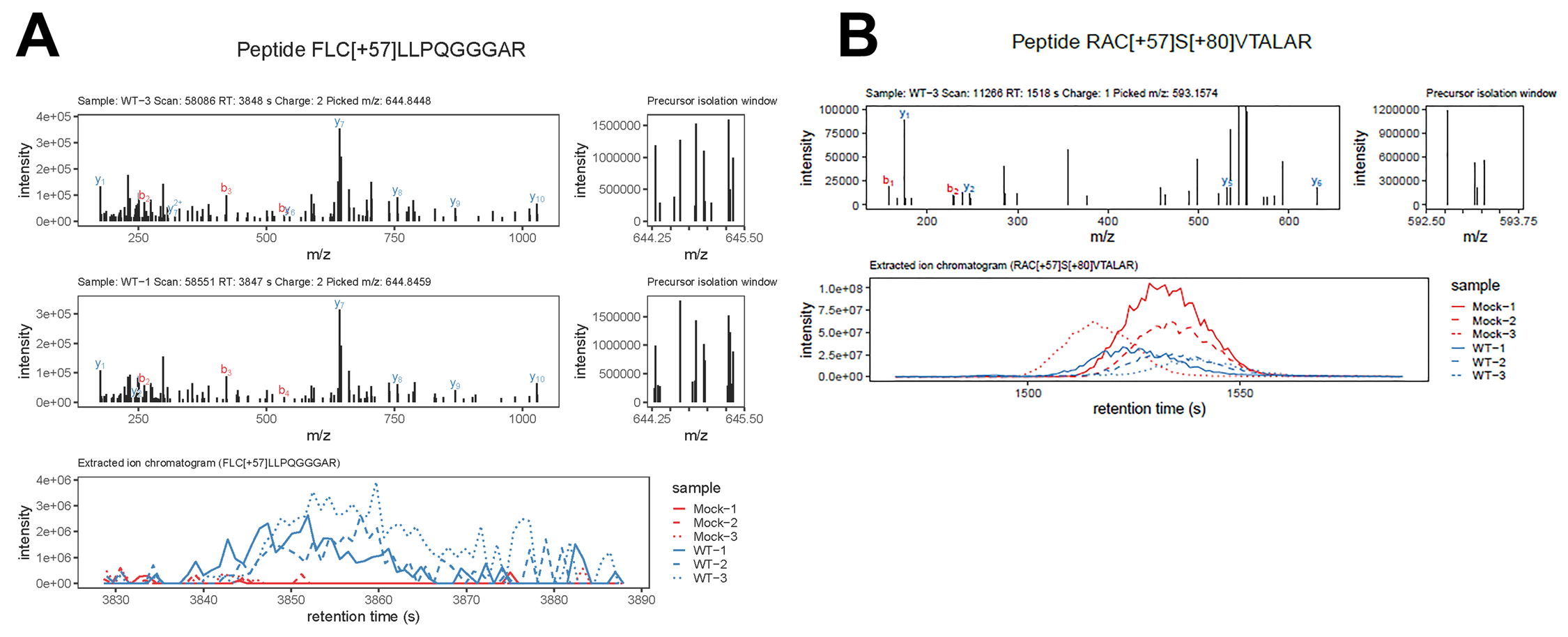

Supplement: S20 Fig — (TIF) [file ppat.1011204.s020.tif]

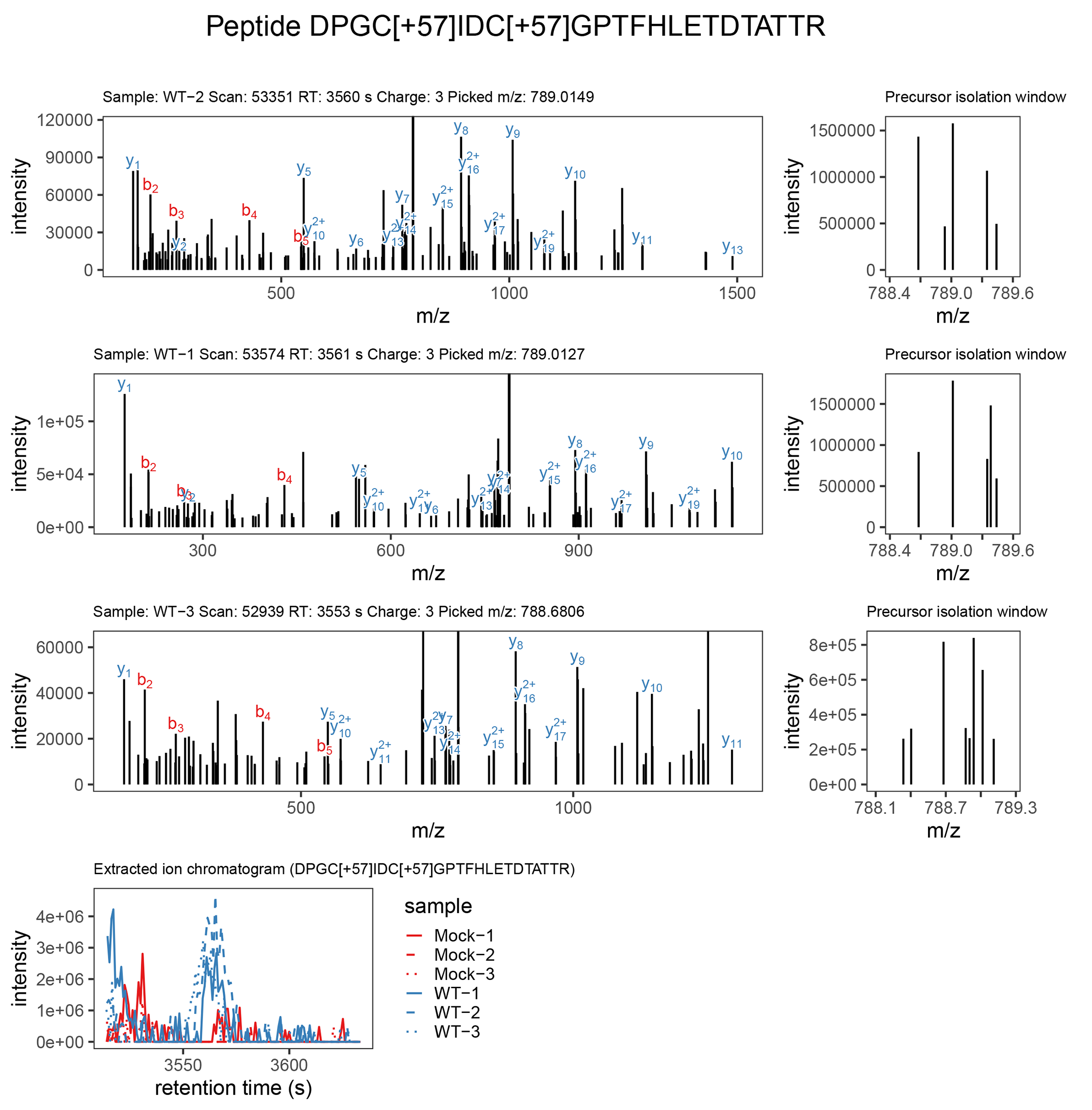

Supplement: S21 Fig — (TIF) [file ppat.1011204.s021.tif]

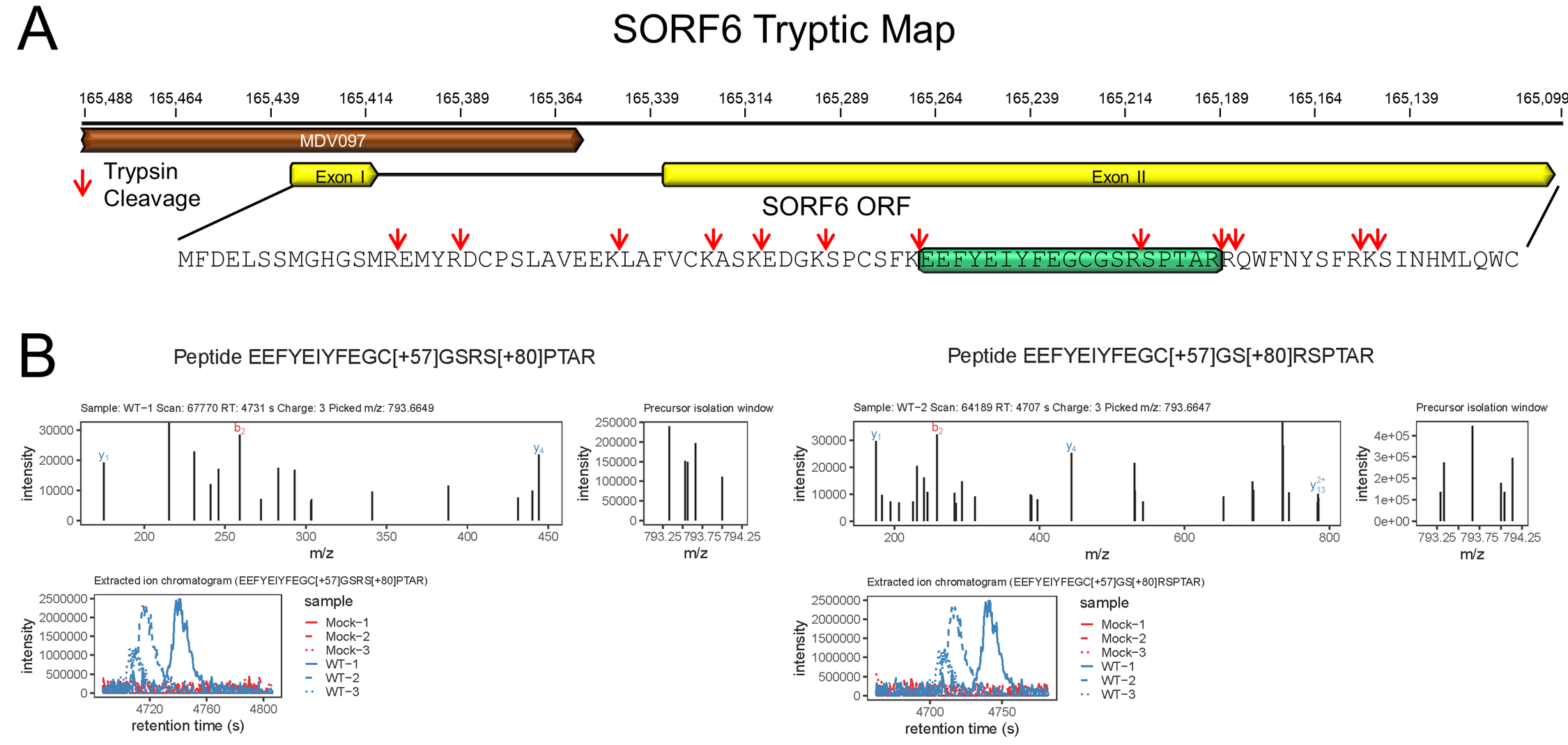

Supplement: S22 Fig — (A) Exon I and II of SORF6, location of tryptic cleavage sites, and peptide identified in infected samples. (B &C) MS2 spectra and six-replicate XIC profiles for the putative SORF6 peptide (the two spectra are assigned to an identical peptide with a phosphorylation localized to different serine residues). (TIF) [file ppat.1011204.s022.tif]

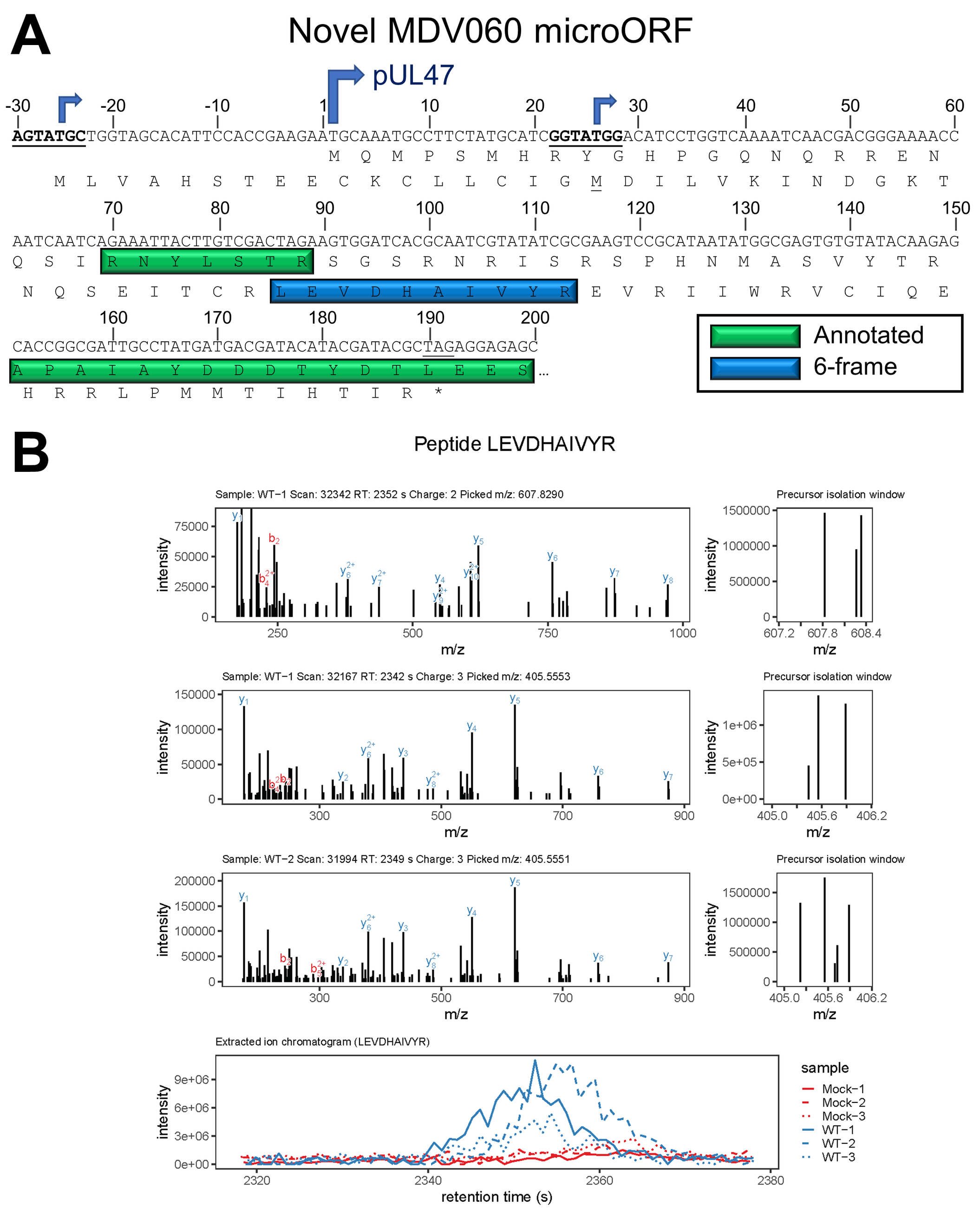

Supplement: S23 Fig — (A) 5’ region of MDV060 with the coding sequence, TIS for pUL47, and identified peptides mapping to annotated pUL47 in green. A novel peptide (blue) was detected using 6-frame translation search mapping to a novel out-of-frame 5’ microORF. Two potential TIS for the novel microORF are shown. (B) For the novel peptide, annotated MS2 scans from the top PSMs showing rich y-ion series and six-replicate XIC showing precursor specificity for infected replicates. (TIF) [file ppat.1011204.s023.tif]

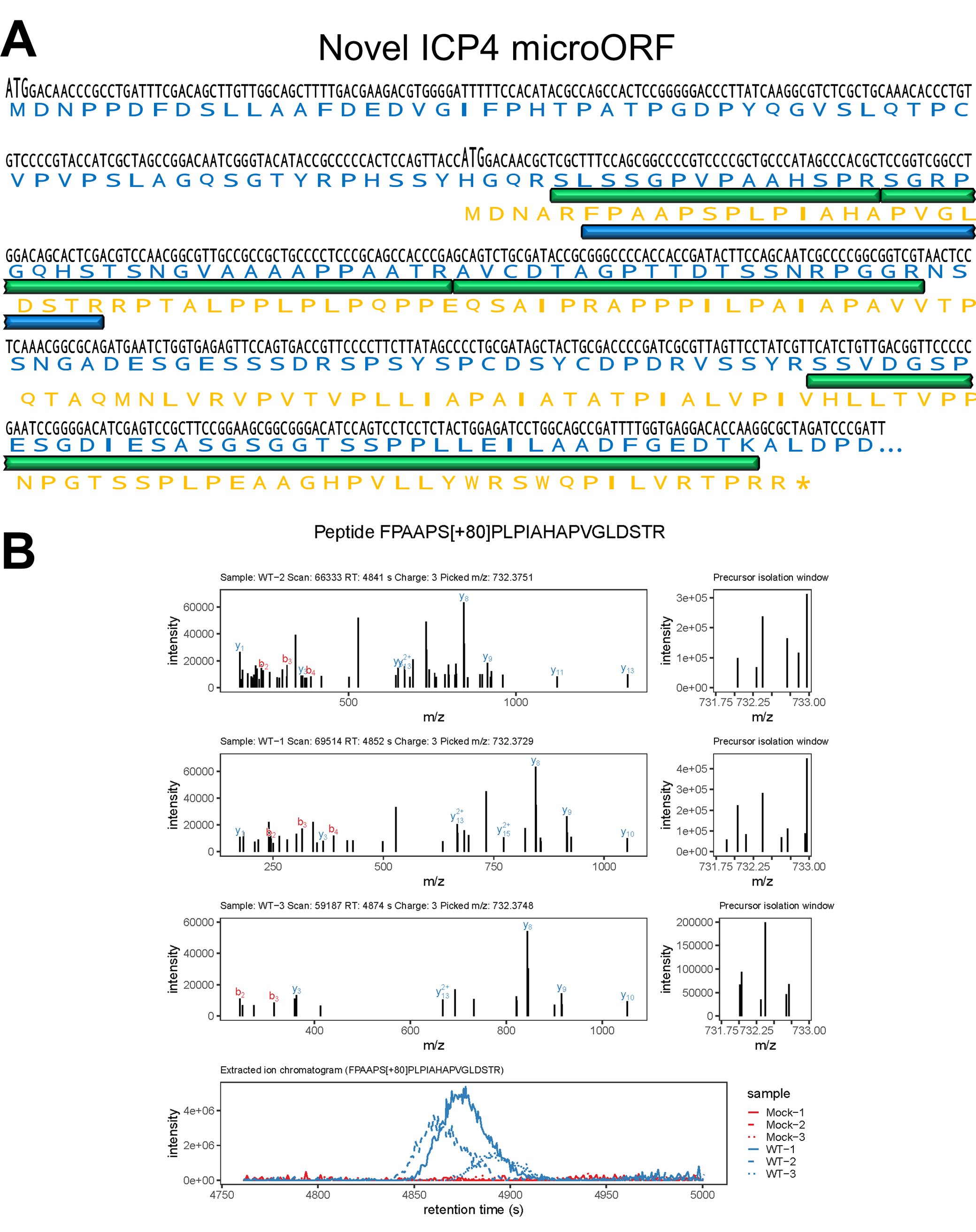

Supplement: S24 Fig — (A) 5’ region of MDV084 with the coding sequence, TIS for ICP4, and identified peptides mapping to annotated ICP4 in green. A novel peptide (blue) was detected using 6-frame translation search mapping to a novel out-of-frame 5’ microORF (orange). B) For the novel peptide, annotated MS2 scans from the top PSMs showing decent y-ion series with strong proline-effect peaks and six-replicate XIC showing precursor specificity for infected replicates. (TIF) [file ppat.1011204.s024.tif]

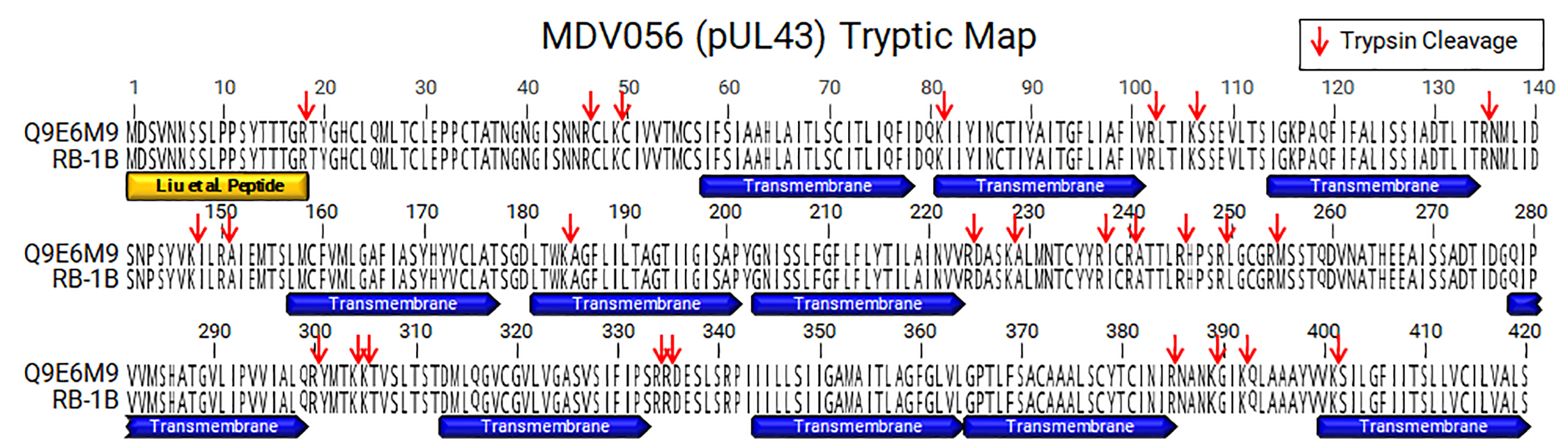

Supplement: S25 Fig — Protein sequence of MDV056 (pUL43) comparing the reference sequence (Q9E6M9) and the RB-1B strain used in this study, plus the predicted tryptic cleavage sites. Transmembrane regions and the unique peptide identified in Liu et al. [23] are shown. (TIF) [file ppat.1011204.s025.tif]

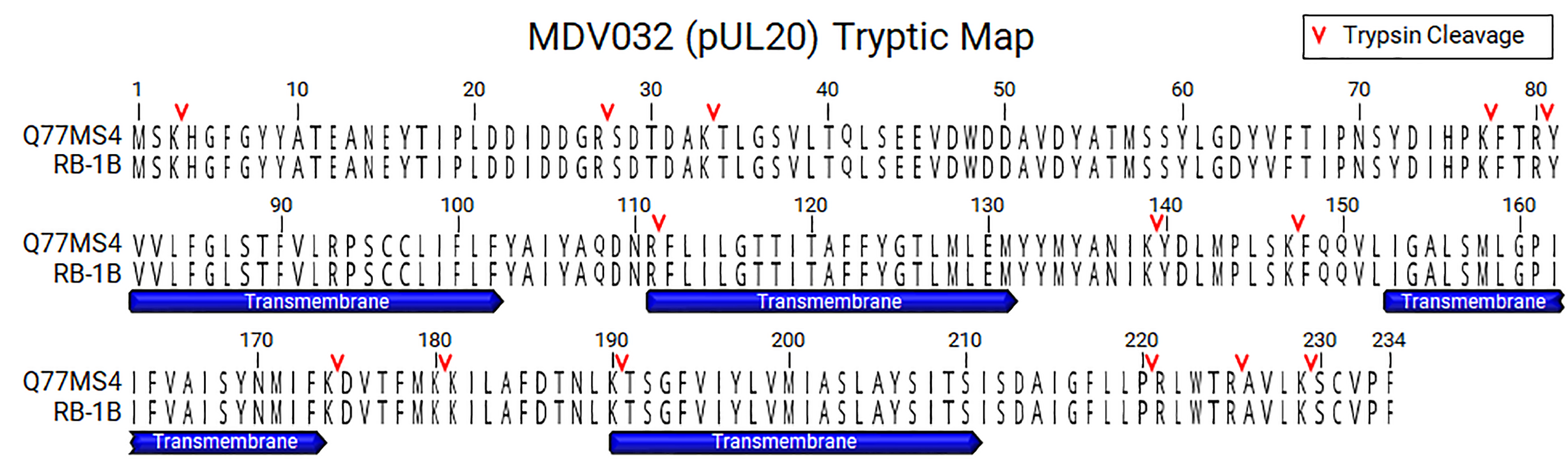

Supplement: S26 Fig — Protein sequence of MDV032 (pUL20) comparing the reference sequence (Q77MS4) and the RB-1B strain used in this study. The predicted tryptic cleavage sites and transmembrane regions are shown. (TIF) [file ppat.1011204.s026.tif]

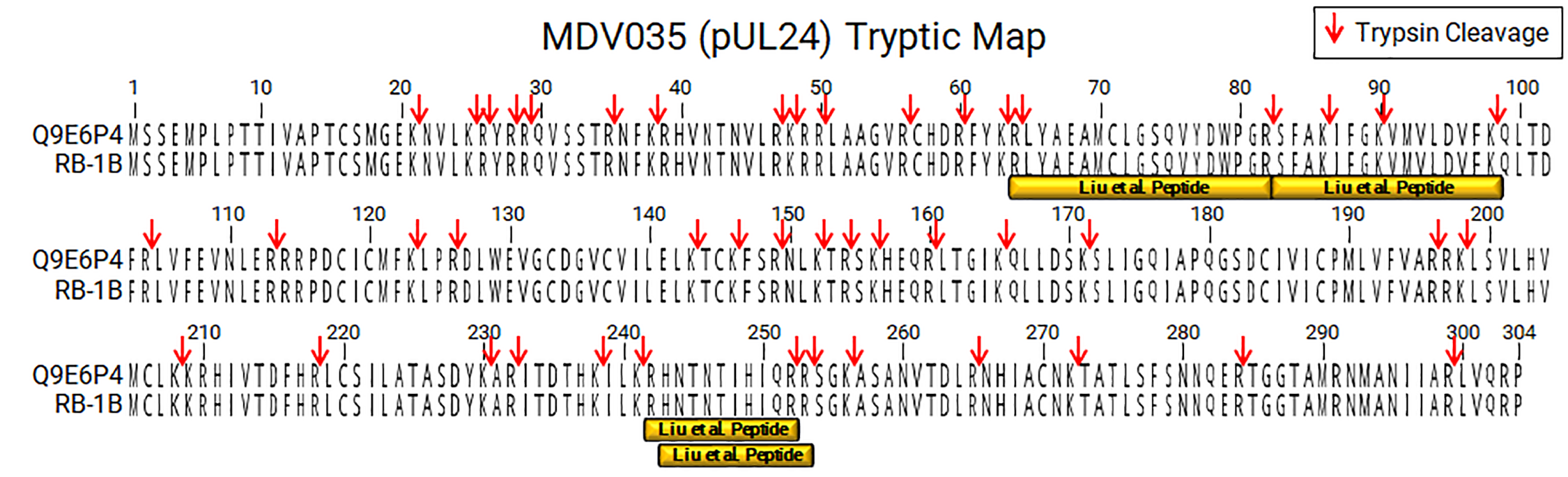

Supplement: S27 Fig — Protein sequence of MDV035 (pUL24) comparing the reference sequence (Q9E6P4) and the RB-1B strain used in this study. The predicted tryptic cleavage sites and unique peptides identified in Liu et al. [23] are shown. (TIF) [file ppat.1011204.s027.tif]

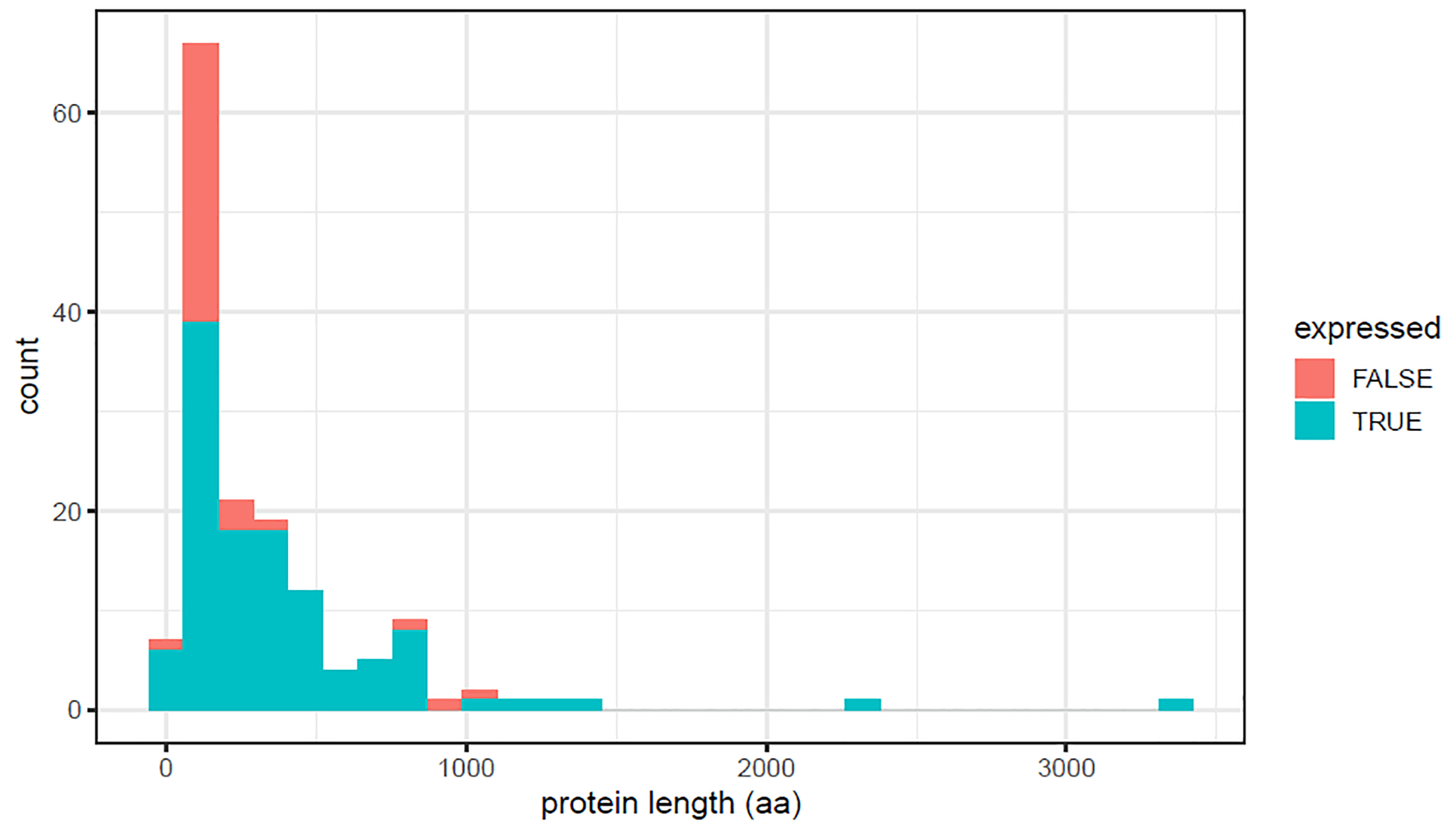

Supplement: S28 Fig — Stacked histogram shows distribution of protein lengths grouped by short-read RNA-Seq expression status (see text for criteria used). (TIF) [file ppat.1011204.s028.tif]
