# Supplementary material for: Viral proteogenomic and expression profiling during productive replication of a skin-tropic herpesvirus in the natural host
Source: PLoS Pathog. 2023 Jun 8;19(6):e1011204. doi: 10.1371/journal.ppat.1011204 (PMC10284419; doi:10.1371/journal.ppat.1011204)
Supplement: S10 Table — (DOCX) [file ppat.1011204.s038.docx]

**S10 Table. Comparison of the current and former reports for RNA sequencing and proteomics during MDV infection in different cell systems.**

|  |  | **RNA** | | | | **Protein** | | | | | |  |
| --- | --- | --- | --- | --- | --- | --- | --- | --- | --- | --- | --- | --- |
| **Master** | **Description (Common Name)** | **CEC^1^** | **B cell^2^** | **FFE^3^** | **FFE^4^** | | **MDCC^5^** | **B cell^2^** | **CEC^6^** | **CEC^7^** | **FFE^4^** | |
| MDV008 | RLORF14 (pp24) | **+** | **+** | **-** | **+** | | **-** | **-** | **+** | **+** | **+** | |
| MDV008.8 | Uncharacterized | **-** | **-** | **-** | **+** | | **-** | **-** | **-** | **-** | **-** | |
| MDV009 | LORF1 | **+** | **+** | **-** | **-** | | **-** | **-** | **+** | **-** | **-** | |
| MDV009.5 | RLORF13 | **+** | **+** | **-** | **+** | | **-** | **-** | **-** | **-** | **-** | |
| MDV010 | Viral lipase-VLIP (LORF2) | **+** | **+** | **+** | **+** | | **-** | **-** | **+** | **-** | **+** | |
| MDV011.5 | Uncharacterized | **+** | **-** | **-** | **-** | | **-** | **-** | **-** | **-** | **-** | |
| MDV012 | p012 | **+** | **+** | **-** | **+** | | **-** | **-** | **+** | **-** | **+** | |
| MDV012.4 | Uncharacterized | **+** | **-** | **-** | **-** | | **-** | **-** | **-** | **-** | **-** | |
| MDV012.8 | Uncharacterized | **+** | **-** | **-** | **+** | | **-** | **-** | **-** | **-** | **-** | |
| MDV013 | UL1-gL (Envelope glycoprotein L) | **+** | **+** | **-** | **+** | | **-** | **-** | **-** | **-** | **+** | |
| MDV013.5 | LORF4 | **-** | **-** | **-** | **-** | | **-** | **-** | **-** | **-** | **-** | |
| MDV014 | UL2-UNG (Uracil-DNA glycosylase) | **+** | **+** | **-** | **+** | | **-** | **+** | **+** | **+** | **+** | |
| MDV014.5 | Uncharacterized | **+** | **-** | **-** | **+** | | **-** | **-** | **-** | **-** | **-** | |
| MDV015 | UL3-NP03 (Nuclear protein 3) | **+** | **+** | **+** | **+** | | **-** | **-** | **+** | **-** | **+** | |
| MDV015.5 | UL3.5-like protein (V57) | **+** | **+** | **-** | **+** | | **-** | **-** | **-** | **-** | **-** | |
| MDV016 | UL4-NP04 (Nuclear protein 4) | **+** | **+** | **+** | **+** | | **-** | **-** | **+^** | **-** | **+** | |
| MDV017 | UL5-HELI (DNA replication helicase) | **+** | **+** | **-** | **-*** | | **-** | **-** | **+** | **-** | **+** | |
| MDV018 | UL6-PORTL (Portal protein) | **+** | **+** | **-** | **+** | | **-** | **-** | **+** | **-** | **+** | |
| MDV019 | UL7-CEP1 (Cytoplasmic envelopment protein 1) | **+** | **+** | **+** | **+** | | **-** | **-** | **-** | **+** | **+** | |
| MDV020 | UL8-HEPA (Helicase/primase-associated protein) | **+** | **+** | **+** | **+** | | **+** | **-** | **+** | **-** | **+** | |
| MDV020.5 | UL8.5-OBPC (Origin-binding protein C) | **-** | **-** | **-** | **+** | | **?** | **-** | **+^±^** | **-** | **+^±^** | |
| MDV021 | UL9-OBP (Ori binding protein) | **+** | **+** | **-** | **+** | | **+** | **-** | **+** | **-** | **+** | |
| MDV022 | UL10-gM (Envelope glycoprotein M) | **+** | **+** | **-** | **+** | | **-** | **-** | **+^** | **-** | **+** | |
| MDV023 | UL11-CEP3 (Cytoplasmic envelopment protein 3) | **-** | **+** | **-** | **+** | | **-** | **-** | **+^** | **-** | **+^** | |
| MDV024 | UL12-AK (Alkaline nuclease) | **+** | **+** | **+** | **+** | | **+** | **-** | **+** | **+** | **+** | |
| MDV025 | UL13-CHPK (Conserved herpesvirus protein kinase) | **+** | **+** | **-** | **+** | | **-** | **-** | **+** | **-** | **+** | |
| MDV026 | UL14-TEG3 (Tegument protein 3) | **+** | **+** | **-** | **+** | | **-** | **-** | **+^** | **-** | **+** | |
| MDV027 | UL15-TRM3 (Tripartite terminase subunit 3) | **+** | **+** | **-** | **+** | | **-** | **-** | **+** | **-** | **+** | |
| MDV028^ | UL16-CEP2 (Cytoplasmic envelopment protein 2) | **+** | **+** | **-** | **+** | | **-** | **-** | **-** | **-** | **+** | |
| MDV029 | UL17-CVC1 (Capsid vertex component) | **+** | **+** | **-** | **+** | | **-** | **-** | **+^** | **-** | **+** | |
| MDV030 | UL18-TRX2 (Triplex capsid protein 2) | **+** | **+** | **+** | **+** | | **-** | **-** | **+** | **+** | **+** | |
| MDV031 | UL19-MCP (Major capsid protein-VP5) | **+** | **+** | **+** | **+** | | **+** | **+** | **+** | **+** | **+** | |
| MDV031.5 | Uncharacterized | **+** | **-** | **-** | **+** | | **-** | **-** | **-** | **-** | **-** | |
| MDV032 | UL20-MP1 (Membrane protein 1) | **+** | **+** | **-** | **+** | | **+** | **-** | **-** | **-** | **-** | |
| MDV033 | UL21-TEG4 (Tegument protein 4) | **+** | **+** | **-** | **+** | | **-** | **-** | **-** | **-** | **+** | |
| MDV034 | UL22-gH (Envelope glycoprotein H) | **+** | **+** | **-** | **+** | | **-** | **-** | **+** | **-** | **+** | |
| MDV035 | UL24-NP24 (nuclear protein UL24) | **+** | **+** | **-** | **+** | | **-** | **-** | **+** | **-** | **-** | |
| MDV036 | UL23-TK (Thymidine kinase) | **+** | **+** | **-** | **+** | | **-** | **+** | **+^** | **+** | **+** | |
| MDV037 | UL25-CVC2 (Capsid vertex component 2) | **+** | **+** | **+** | **+** | | **-** | **-** | **+** | **-** | **+** | |
| MDV038 | UL26-SCAF (Capsid scaffolding protein) | **+** | **+** | **+** | **+** | | **-** | **+** | **+** | **+** | **+** | |
| MDV038.5 | Uncharacterized (C2H2 domain protein) | **-** | **-** | **-** | **+** | | **-** | **-** | **-** | **-** | **-** | |
| MDV039 | UL26.5-ICP35 (Capsid scaffold protein) | **+** | **+** | **+** | **+** | | **-** | **+^±^** | **+^±^** | **-** | **+^@^** | |
| MDV039.5 | LORF5 | **-** | **-** | **-** | **-** | | **-** | **-** | **-** | **-** | **-** | |
| MDV040 | UL27-gB (Envelope glycoprotein B) | **+** | **+** | **+** | **+** | | **-** | **-** | **+** | **-** | **+** | |
| MDV041 | UL28-TRM1 (Tripartite terminase subunit 1) | **+** | **+** | **-** | **+** | | **-** | **-** | **+** | **-** | **+** | |
| MDV042 | UL29-DNBI (Major DNA-binding protein) | **+** | **+** | **-** | **+** | | **-** | **+** | **+** | **+** | **+** | |
| MDV043 | UL30-DPOL (DNA polymerase ) | **+** | **+** | **-** | **+** | | **-** | **-** | **+** | **-** | **+** | |
| MDV044 | UL31-NEC1 (Nuclear egress protein 1) | **+** | **+** | **-** | **+** | | **-** | **-** | **+^** | **-** | **+** | |
| MDV045 | UL33-TRM2 (Tripartite terminase subunit 2) | **+** | **+** | **-** | **+** | | **-** | **-** | **+** | **-** | **+** | |
| MDV046 | UL32 (Packaging protein UL32) | **+** | **+** | **-** | **+** | | **+** | **-** | **+** | **+** | **+** | |
| MDV047 | UL34-NEC2 (Nuclear egress protein 2) | **+** | **+** | **+** | **+** | | **-** | **+** | **+** | **+** | **+** | |
| MDV048 | UL35-SCP (Small capsomere-interacting protein) | **+** | **+** | **-** | **+** | | **-** | **-** | **-** | **-** | **+** | |
| MDV049 | UL36-LTP (Large tegument protein deneddylase) | **+** | **+** | **+** | **+** | | **+** | **-** | **+** | **-** | **+** | |
| MDV049.5 | LORF6 | **-** | **-** | **-** | **-** | | **-** | **-** | **-** | **-** | **-** | |
| MDV050 | UL37-ITP (Inner tegument protein) | **+** | **+** | **-** | **+** | | **+** | **-** | **+** | **-** | **+** | |
| MDV050.5 | LORF7 | **-** | **-** | **-** | **-** | | **-** | **-** | **-** | **-** | **-** | |
| MDV051 | UL38-TRX1 (Triplex capsid protein 1) | **+** | **+** | **-** | **+** | | **+** | **-** | **+^** | **-** | **+** | |
| MDV052 | UL39-RIR1 (rNDP large subunit) | **+** | **+** | **-** | **+** | | **+** | **+** | **+** | **+** | **+** | |
| MDV053 | UL40-RIR2 (rNDP small subunit) | **+** | **+** | **-** | **+** | | **+** | **+** | **+** | **+** | **+** | |
| MDV054* | UL41-SHUT (Virion host shutoff protein) | **+** | **+** | **-** | **+** | | **-** | **-** | **+^** | **-** | **+** | |
| MDV055 | UL42-PAP (DNA polymerase processivity factor) | **+** | **+** | **-** | **+** | | **-** | **+** | **+** | **+** | **+** | |
| MDV056 | UL43-MB43 (Membrane protein) | **+** | **+** | **-** | **+** | | **-** | **-** | **+^** | **-** | **-** | |
| MDV057 | UL44-gC (Envelope glycoprotein C or A antigen) | **+** | **+** | **-** | **+** | | **+** | **-** | **+** | **+** | **+** | |
| MDV057.4 | Uncharacterized | **+** | **-** | **-** | **+** | | **-** | **-** | **-** | **-** | **-** | |
| MDV057.8 | LORF8 (23 kDa protein) | **+** | **-** | **-** | **-** | | **-** | **-** | **+** | **-** | **-** | |
| MDV058 | UL45-EV45 (Envelope protein) | **+** | **+** | **-** | **+** | | **-** | **-** | **-** | **-** | **+** | |
| MDV059 | UL46-TEG1 (Tegument protein 1-VP11/12) | **+** | **+** | **+** | **+** | | **+** | **-** | **+** | **-** | **+** | |
| MDV060 | UL47-TEG5 (Tegument protein 5-VP13/14) | **+** | **+** | **-** | **+** | | **-** | **-** | **+** | **-** | **+** | |
| MDV061 | UL48-VP16 (Alpha trans-inducing protein-VP16) | **+** | **+** | **-** | **+** | | **-** | **-** | **+^** | **-** | **+** | |
| MDV062 | UL49-VP22 (Tegument protein-VP22) | **+** | **+** | **-** | **+** | | **+** | **+** | **+** | **+** | **+** | |
| MDV063 | UL50-DUT (dUTP nucleotidohydrolase) | **+** | **+** | **-** | **+** | | **-** | **+** | **+** | **+** | **+** | |
| MDV063.5 | Uncharacterized | **-** | **-** | **-** | **+** | | **-** | **-** | **-** | **-** | **-** | |
| MDV064 | UL49.5-gN (Envelope glycoprotein N) | **+** | **+** | **-** | **+** | | **-** | **-** | **-** | **-** | **+** | |
| MDV065 | UL51-TEG7 (Tegument protein 7) | **+** | **+** | **-** | **+** | | **-** | **-** | **+^** | **+** | **+** | |
| MDV066 | UL52-PRIM (DNA primase) | **+** | **+** | **-** | **-*** | | **-** | **-** | **+** | **-** | **+** | |
| MDV067 | UL53-gK (Envelope glycoprotein K) | **+** | **+** | **+** | **+** | | **-** | **-** | **-** | **-** | **+** | |
| MDV068 | UL54-ICP27 (mRNA export factor ICP27) | **+** | **+** | **+** | **+** | | **-** | **-** | **+** | **-** | **+** | |
| MDV069 | LORF9 | **+** | **+** | **-** | **+** | | **-** | **-** | **+^** | **-** | **+** | |
| MDV070 | UL55-TEG6 (Tegument protein 6) | **+** | **+** | **-** | **+** | | **-** | **-** | **+^** | **-** | **+** | |
| MDV071 | LORF10 | **-** | **+** | **+** | **+** | | **-** | **-** | **+^** | **-** | **+** | |
| MDV071.4 | Uncharacterized | **-** | **-** | **-** | **-** | | **-** | **-** | **-** | **-** | **-** | |
| MDV072 | LORF11 | **+** | **+** | **-** | **-*** | | **-** | **-** | **+** | **-** | **+*** | |
| MDV072.2 | Uncharacterized | **-** | **-** | **-** | **-** | | **-** | **-** | **-** | **-** | **-** | |
| MDV072.4 | Uncharacterized | **-** | **-** | **-** | **-** | | **-** | **-** | **-** | **-** | **-** | |
| MDV072.6 | Uncharacterized | **-** | **-** | **-** | **-** | | **-** | **-** | **-** | **-** | **-** | |
| MDV072.8 | UL56 (LORF12) | **-** | **+** | **-** | **+** | | **-** | **-** | **+** | **-** | **-** | |
| MDV073 | RLORF14a (phosphoprotein 38-pp38) | **+** | **+** | **-** | **+** | | **-** | **-** | **+^** | **+** | **+** | |
| MDV073.4 | RLORF13a | **+** | **-** | **-** | **+** | | **-** | **-** | **+** | **-** | **-** | |
| MDV074 | RLORF12 | **+** | **-** | **-** | **-** | | **-** | **-** | **+^** | **-** | **-** | |
| MDV075 | 14 kDa lytic phase protein A (RLOR13a) | **+** | ***** | **-** | **+** | | **-** | **-** | **+^±^** | **-** | **+** | |
| MDV075.1 | B68 (VZV transinducing protein) | **-** | **-** | **-** | **+** | | **-** | **-** | **-** | **-** | **-** | |
| MDV075.2 | Uncharacterized | **+** | **-** | **-** | **+** | | **-** | **-** | **-** | **-** | **+** | |
| MDV075.3 | 14 kDa lytic phase protein B | **-** | **+** | **-** | **+** | | **-** | **-** | **+^±^** | **-** | **+^±^** | |
| MDV075.4 | RLORF11 | **+** | **-** | **-** | **-** | | **-** | **-** | **+** | **-** | **-** | |
| MDV075.5 | Uncharacterized | **-** | **-** | **-** | **+** | | **-** | **-** | **-** | **-** | **-** | |
| MDV075.6 | Uncharacterized | **-** | **-** | **-** | **+** | | **-** | **-** | **-** | **-** | **+** | |
| MDV075.7 | RLORF10 | **+** | **-** | **-** | **-** | | **-** | **-** | **-** | **-** | **-** | |
| MDV075.8 | RLORF9 (14-kDa lytic protein C) | **+** | **-** | **-** | **+** | | **+** | **-** | **+** | **-** | **-** | |
| MDV075.9 | Uncharacterized | **+** | **-** | **-** | **+** | | **-** | **-** | **-** | **-** | **-** | |
| MDV075.91 | Uncharacterized | **-** | **-** | **-** | **-** | | **-** | **-** | **-** | **-** | **-** | |
| MDV075.92 | RLORF8 | **+** | **-** | **-** | **+** | | **-** | **-** | **-** | **-** | **-** | |
| MDV076 | RLORF7 (Oncogene Meq) | **+** | **+** | **+** | **+** | | **-** | **-** | **-** | **-** | **+^*** | |
| MDV076.4 | Uncharacterized | **+** | **-** | **-** | **+** | | **-** | **-** | **-** | **-** | **-** | |
| MDV076.8 | Uncharacterized | **+** | **-** | **-** | **-** | | **-** | **-** | **-** | **-** | **-** | |
| MDV077 | 23 kD nuclear protein | **+** | **+** | **-** | **-** | | **-** | **-** | **+** | **-** | **-** | |
| MDV077.5 | RLORF6 | **-** | **-** | **-** | **+** | | **-** | **-** | **+^** | **-** | **-** | |
| MDV078 | Viral CXCvIL8 homolog | **+** | **+** | **-** | **+** | | **-** | **-** | **+^** | **-** | **+** | |
| MDV078.1 | RLORF5a – L1 Protein | **+** | **-** | **-** | **+** | | **-** | **-** | **-** | **-** | **+** | |
| MDV078.2 | RLORF5 | **-** | **-** | **-** | **-** | | **+** | **-** | **-** | **-** | **-** | |
| MDV078.3 | RLORF4 | **+** | **+** | **-** | **-** | | **+** | **-** | **+** | **-** | **-** | |
| MDV078.4 | RLORF3 | **+** | **-** | **-** | **+** | | **-** | **-** | **-** | **-** | **-** | |
| MDV078.5 | Uncharacterized | **+** | **-** | **-** | **+** | | **-** | **-** | **-** | **-** | **-** | |
| MDV078.6 | Uncharacterized | **-** | **-** | **-** | **+** | | **-** | **-** | **-** | **-** | **-** | |
| MDV079 | RLORF1 | **-** | **-** | **-** | **+** | | **-** | **-** | **+** | **-** | **-** | |
| MDV080 | Uncharacterized | **-** | **+** | **-** | **+** | | **-** | **-** | **-** | **-** | **-** | |
| MDV080.5 | Uncharacterized | **-** | **-** | **-** | **-** | | **-** | **-** | **-** | **-** | **-** | |
| MDV081 | Uncharacterized | **+** | **+** | **-** | **+** | | **-** | **-** | **-** | **-** | **-** | |
| MDV081.5 | Uncharacterized | **-** | **-** | **-** | **+** | | **-** | **-** | **-** | **-** | **-** | |
| MDV082 | RSORF1 | **+** | **+** | **-** | **+** | | **-** | **-** | **+** | **+** | **+** | |
| MDV083 | Antisense RNA protein | **+** | **+** | **-** | **+** | | **-** | **-** | **+** | **-** | **-** | |
| MDV084 | Major viral transcription factor ICP4 | **+** | **-** | **-** | **+** | | **+** | **+** | **+** | **-** | **+** | |
| MDV084.5 | Uncharacterized | **+** | **-** | **-** | **-** | | **-** | **-** | **-** | **-** | **-** | |
| MDV085 | Uncharacterized | **+** | **+** | **-** | **-** | | **-** | **-** | **+** | **-** | **-** | |
| MDV085.3 | Uncharacterized | **+** | **-** | **-** | **+** | | **-** | **-** | **-** | **-** | **-** | |
| MDV085.6 | Uncharacterized | **+** | **-** | **-** | **-** | | **-** | **-** | **-** | **-** | **-** | |
| MDV085.9 | Uncharacterized | **+** | **-** | **-** | **-** | | **-** | **-** | **-** | **-** | **-** | |
| MDV086 | Uncharacterized | **-** | **+** | **-** | **+** | | **-** | **-** | **+** | **-** | **-** | |
| MDV086.1 | Uncharacterized | **+** | **-** | **-** | **+** | | **-** | **-** | **-** | **-** | **-** | |
| MDV086.2 | Uncharacterized | **-** | **-** | **-** | **-** | | **-** | **-** | **-** | **-** | **-** | |
| MDV086.4 | Uncharacterized | **-** | **-** | **-** | **-** | | **-** | **-** | **-** | **-** | **-** | |
| MDV086.6 | SORF1 | **+** | **-** | **-** | **-** | | **-** | **-** | **-** | **-** | **-** | |
| MDV087 | SORF2 (Growth hormone-binding protein) | **+** | **+** | **-** | **+** | | **-** | **-** | **+** | **+** | **+** | |
| MDV088 | US1-ICP22 (Immediate early protein ICP22) | **+** | **+** | **-** | **+** | | **+** | **-** | **+** | **-** | **+** | |
| MDV089 | US10 (Virion protein) | **+** | **+** | **+** | **+** | | **-** | **-** | **+** | **-** | **+** | |
| MDV089.5 | 14 kD protein (uncharacterized) | **+** | **-** | **-** | **-** | | **-** | **-** | **-** | **-** | **-** | |
| MDV090 | SORF3 | **+** | **+** | **+** | **+** | | **-** | **-** | **+^** | **-** | **+** | |
| MDV091 | US2 | **+** | **+** | **-** | **+** | | **+** | **-** | **+** | **-** | **+** | |
| MDV091.5 | Uncharacterized | **-** | **-** | **-** | **-** | | **-** | **-** | **-** | **-** | **+^** | |
| MDV092 | US3 (Protein kinase) | **+** | **+** | **-** | **+** | | **+** | **-** | **+** | **-** | **+** | |
| MDV092.4 | Uncharacterized | **+** | **-** | **-** | **-** | | **-** | **-** | **-** | **-** | **-** | |
| MDV092.8 | Uncharacterized | **+** | **-** | **-** | **-** | | **-** | **-** | **-** | **-** | **-** | |
| MDV093 | SORF4 | **+** | **+** | **+** | **+** | | **-** | **-** | **+^** | **-** | **+^** | |
| MDV094 | US6-gD (Envelope glycoprotein D) | **+** | **+** | **-** | **+** | | **-** | **-** | **-** | **-** | **+** | |
| MDV094.5 | Uncharacterized (US426) | **+** | **-** | **-** | **-** | | **-** | **-** | **-** | **-** | **-** | |
| MDV095 | US7-gI (Envelope glycoprotein I) | **+** | **+** | **+** | **+** | | **+** | **-** | **+** | **-** | **+** | |
| MDV095.5 | Uncharacterized | **+** | **-** | **-** | **+** | | **-** | **-** | **-** | **-** | **-** | |
| MDV096 | US8-gE (Envelope glycoprotein E) | **+** | **+** | **+** | **+** | | **+** | **-** | **+** | **+** | **+** | |
| MDV096.5 | Uncharacterized | **-** | **-** | **-** | **+** | | **-** | **-** | **-** | **-** | **-** | |
| MDV097 | SORF2A | **-** | **-** | **-** | **+** | | **-** | **-** | **-** | **+** | **-** | |
| MDV097.3 | Uncharacterized | **-** | **-** | **-** | **-** | | **-** | **-** | **-** | **-** | **-** | |
| MDV097.6 | Uncharacterized | **-** | **-** | **-** | **-** | | **-** | **-** | **-** | **-** | **-** | |

^One unique peptide detected.

*Protein identified in phosho-enriched sample. Absent in global peptide analysis.

±Peptides duplicated with other proteins.

^@^Confirmed by unique translational initiation site peptide.

**References**

^1^Sadigh Y, Tahiri-Alaoui A, Spatz S, Nair V, Ribeca P. Pervasive Differential Splicing in Marek's Disease Virus can Discriminate CVI-988 Vaccine Strain from RB-1B Very Virulent Strain in Chicken Embryonic Fibroblasts. Viruses. 2020;12(3). doi: 10.3390/v12030329.

^2^ Bertzbach LD, Pfaff F, Pauker VI, Kheimar AM, Hoper D, Hartle S, et al. The Transcriptional Landscape of Marek's Disease Virus in Primary Chicken B Cells Reveals Novel Splice Variants and Genes. Viruses. 2019;11(3). doi: 10.3390/v11030264.

^3^Sunkaraa L, Ahmad SM, Heidari M. RNA-seq analysis of viral gene expression in the skin of Marek's disease virus infected chickens. Vet Immunol Immunopathol. 2019;213:109882. doi: 10.1016/j.vetimm.2019.109882.

^4^This report.

^5^Buza JJ, Burgess SC. Modeling the proteome of a Marek's disease transformed cell line: a natural animal model for CD30 overexpressing lymphomas. Proteomics. 2007;7(8):1316-26. doi: 10.1002/pmic.200600946.

^6^Liu HC, Soderblom EJ, Goshe MB. A mass spectrometry-based proteomic approach to study Marek's Disease Virus gene expression. J Virol Methods. 2006;135(1):66-75. doi: 10.1016/j.jviromet.2006.02.001.

^7^Chien KY, Blackburn K, Liu HC, Goshe MB. Proteomic and phosphoproteomic analysis of chicken embryo fibroblasts infected with cell culture-attenuated and vaccine strains of Marek's disease virus. J Proteome Res. 2012;11(12):5663-77. doi: 10.1021/pr300471y. PubMed PMID: 23106611
