# Supplementary material for: Viral proteogenomic and expression profiling during productive replication of a skin-tropic herpesvirus in the natural host
Source: PLoS Pathog. 2023 Jun 8;19(6):e1011204. doi: 10.1371/journal.ppat.1011204 (PMC10284419; doi:10.1371/journal.ppat.1011204)
Supplement: Attachment — Submitted filename: PPATHOGENS-D-23-00283 Rebuttal.pdf [file ppat.1011204.s040.pdf]

PPATHOGENS-D-23-00283

Response to Reviewer Comments (responses in **blue**)

Viral proteogenomic and expression profiling during fully productive replication of a skin-tropic herpesvirus in the natural host

Thank you very much for submitting your manuscript "Viral proteogenomic and expression profiling during fully productive replication of a skin-tropic herpesvirus in the natural host" for consideration at PLOS Pathogens. As with all papers reviewed by the journal, your manuscript was reviewed by members of the editorial board and by several independent reviewers. While each of the reviewers were impressed with the scope of work done, they also pointed out several weaknesses in the current version. This includes the perception of a lack of novelty in the approach and the descriptive nature of the studies presented. Reviewers suggested that some of these weaknesses could be mitigated with a more extensive and in depth re-write. Reviewers also noted that two additional experiments would further strengthen the manuscript experimentally and should be included. Please be sure to address all of the reviewers comments in the revised version of the manuscript.

Reviewer Responses to Questions

Part I – Summary

Please use this section to discuss strengths/weaknesses of study, novelty/significance, general execution and scholarship.

Reviewer #1: This paper by Volkening et al. describes the proteome and stable transcriptome of Marek's disease virus (MDV) in the skin of chickens. The skin is of interest here because it is the only place in the animal that allows production of infectious virus despite systemic infection. In this way it is reminiscent of Varicella Zoster Virus, another alpha herpesvirus, that replicates in skin cells but is otherwise restricted.

Regarding the significance of the study, there have been several -omic type descriptive papers on MDV recently focusing on either RNA or protein in cultured cells or B cells, but this is the first to combine these in a single set of analyses in a cell type fully permissive to infectious virus production in vivo. The data is extensive, well presented, and will likely represent the go-to data set for MDV gene and protein expression. Although there were relatively few surprises beyond what is expected from annotated genes some areas of interest that have been confusing from the annotation are clarified, including alternative splicing in the abundantly expressed gC and 14Kda loci.

Given the lack of surprises and what appear to be largely similar results from the previous studies, the novelty of the data is not particularly high, although the studies are performed thoroughly and data curated and presented well. A comparative table might be useful to highlight results that are different between the current and previous work.

Authors' response: We agree that a table specifically comparing the current results with those of previous work would be beneficial for the reader and have compiled and included this table as S10_Table.

Reviewer #2: In this manuscript, the authors assessed the transcriptome and proteome of the highly oncogenic Marek's disease virus (MDV) in feather follicle epithelia cells (FFE). The FFE is particular important as this is the only site in animals, where infectious cell free MDV is produced and shed into the environment, which ensure the spread of the virus. Until now, both the transcriptome and proteome in this crucial site remained elusive. This study provides a comprehensive overview and includes a plethora of information that are exciting to the field (as well as other alphaherpesviruses such as VZV) and will provide the basis for many future studies. Overall, the manuscript is well written but is also very technical due to the nature of transcriptome and proteome analyses. Only the following points should be addressed prior to publication.

Authors' response: Thank you for these kind comments. Specific points are addressed below.

Reviewer #3: Volkening et al have generated detailed maps of the transcriptome and proteome of Marek's disease virus (MDV) in feather follicle epithelial skin cells of live chickens. Their powerful approach provides the ability to link viral transcription and protein production in the most relevant system available (live chickens). The analysis presented is thorough and without doubt this work further enhances our knowledge of MDV biology. However, while the level of detail is high, the overarching purpose and results of this study are somewhat lost in the writing (i.e. it is hard to see the wood for the trees). It is thus a very descriptive work which lacks wider context. There also appears to be some missed opportunities to obtain data that would really push this work to the level it deserves. With that in mind, I offer the following critiques and recommended revisions which I consider essential for this work to be accepted.

Authors' response: Thank you for your review. We believe the revised manuscript has addressed these issues and specific points are addressed below.

Part II – Major Issues: Key Experiments Required for Acceptance
Please use this section to detail the key new experiments or modifications of existing experiments that should be absolutely required to validate study conclusions.

Generally, there should be no more than 3 such required experiments or major modifications for a "Major Revision" recommendation. If more than 3 experiments are necessary to validate the study conclusions, then you are encouraged to recommend "Reject".

Reviewer #1: Data is thorough.

Reviewer #2: (No Response)

Reviewer #3: 1. On reading the abstract and introduction, I remain uncertain as to the purpose of the study. While there is obvious value in looking at the viral transcriptome and proteome in this system, the authors do not convey this well, nor do they provide any real comparative analysis against cell culture models to show why their system is so valuable.

Authors' response: Thank you for the feedback regarding a lack of conveyed purpose in the manuscript. We have made extensive edits to the Results/Discussion section to attempt to address more directly what we see as the impact of the current work. Specifically: S10_Table we have also included an additional table directly comparing the results of this work to those of previous related studies.

We have revised the concluding sentences in the introduction to better reflect the importance of this study (lines 101-107).

2. Numerous recent studies have demonstrated the major advantages of long-read sequencing approaches in generate ultra-high resolution maps of viral transcriptomes. While input requirements for some of these (e.g. PacBio, Nanopore Direct RNA-Sequencing, Direct cDNA-Sequencing) are problematic for non-cell culture based systems, approaches such as Nanopore PCR-cDNA sequencing should be compatible. Alternatively, the authors could apply a long-read approach to a cell culture based system to at least characterize the full diversity of the MDV transcriptome and then integrate the RNA-Seq and proteome data from the feather follicle system to look at differences in expression level. While the optimal approach would be to include long-read sequencing datasets for both the feather follicle and a cell culture based system, even a single dataset from the latter could be sufficient to provide a much more detailed analysis including the identification of the boundaries of all encoded mRNAs. This would also significantly enhance the global analysis of splicing patterns across the MDV transcriptome.

Authors' response: We fully agree as to the advantages of long-read sequencing in the context of the questions being addressed here. We had already planned to perform additional combined short- and long-read sequencing under multiple conditions as part of future work. However, we decided to complete sequencing and analysis of the wildtype MDV infection in FFE, in two infected replicates plus one mock replicate, for inclusion in the current manuscript at the reviewer's suggestion. A primary advantage of the selection and isolation methods described is the significant enrichment for viral RNA *in vivo*, and we were able to obtain high depth and breadth of coverage of the MDV transcriptome based on rarefaction analysis. As expected, this resulted in significant additions and revisions to the manuscript. Major changes are noted here:

Updating of Fig 1, Fig 2 (now Fig 7), Figs 4-6 (now 10-11), S1-4 Figs (now Figs 2-6),

Addition of IsoSeq data including S8-S14 Figs, S5-6 Tables

Text in revised MS: Lines 240-433; 455-476; 590-594; 702-704; 743-747; 750-764; 787-792; 861-869; 892-958; 1033-1036

3. From a transcription perspective, figures such as S1-S4 would be valuable to include within the main text itself. It is also confusing to see tracks named as 'genes' and 'introns' rather than having transcript structures (e.g. mRNAs) represented. Similarly, the use of a log2 scale on the coverage plots actually makes it harder to discern regions with abundant transcription from regions with low-level transcription.

Author's response: As the reviewer may imagine, it is a challenge to plot multiple detailed genomic tracks in a way that is both visually informative and suitable for publication in a page-based, size-restricted format. This issue was only exacerbated by the addition of an additional track to show IsoSeq transcripts. We have re-worded and expanded the figure legends to clarify what each track

represents. We believe that the reviewer's suggestions as to track representation and the use of the log2 scale, while reasonable, are matters of opinion which would not be universally shared by all readers. In particular, the choice of scale is necessarily a balance between emphasizing highly expressed transcripts and showing any useful information about less abundant transcripts. Use of a non-log scale would emphasize the former but show little or no useful information about the latter. We would also like to note that the manuscript includes reference to an online IGV-based browser which, in addition to showing all of the tracks in the static figures, also offers additional tracks including non-transformed depth plots. We hope that this will allow readers to visualize the data from the publication in the manner they find easiest to interpret. We have also included in the submitted revisions, as a supplemental file, all BED, GFF, and bedgraph tracks used to produce the genome maps so readers may explore them using their own method of choice.

We have not included former S1-S4 Figs into the main manuscript as Figs 2-6. We created two figures for the UL region as it is much larger than the other regions for readability.

4. The observation regarding lower strand-specificity of the reads aligning to the viral genome when compared to the host bears further investigation. It is not clear from the results/methods how this value was calculated but it seems likely to be an artefact of the analysis rather than a real biological phenomenon. Moreover, the calculation might be biased by the decision to remove terminal repeats from the genome (as specified in the methods). The authors should consider determining the strand-specificity values for the UL and US regions alone to determine whether the observed 66-75% is consistent across the genome. Finally, the analysis of the high strand-specificity observed on the host genome should be shown (rather than just reported as unpublished observation).

Authors' response: We agree that these observations warranted both further investigation and a better description in the manuscript, much of which was left out originally due to length concerns. We re-calculated library strand specificity for both host and virus using only high-confidence regions (chromosome 1 for chicken, unique long region for host). We have included visual representation of this in S5 Fig of the revised MS.

In addition, we used the long-read-derived transcript data as a more complete and accurate annotation for input to the calculation software. These changes significantly increased the calculated strand specificity for viral reads, from 66-75% originally reported up to 84-88%, and the manuscript (lines 180-205) and supplemental S4 Table were updated accordingly. These values are still 9-13% lower than the corresponding host-based calculations (range 95-98%), in agreement with the antisense read mapping that can be seen in the associated genome tracks.

We have added a supplemental S5 Fig for visualizing the sense and antisense read mapping for representative host and viral genes and removed the "unpublished observation" reference.

It is not immediately clear how removal of the terminal repeats for analysis might affect calculation of strand specificity, as the sequences removed were (and are for nearly all published alphaherpesvirus genomes) identical to those of the included internal repeats. As explained in the manuscript, sufficient terminal repeat sequence was retained at the junctions to allow mapping of reads from transcripts occurring across the TRL/UL and US/TRS junctions. No changes have been made to the MS.

5. The observation that the most abundant transcript (B68) is not obviously translated is very interesting. There are various softwares available that can predict whether an RNA is likely to be coding

or non-coding. Further analysis, potentially including some in situ hybridization to see where the RNA localizes would seem warranted here.

Authors' response: Thank you for this comment. The IsoSeq data shed additional light on this locus, and we have updated the results and discussion accordingly (lines 206-221 of revised MS). The unspliced transcript with B68 as the most upstream ORF was (by far) also the most highly expressed transcript in the IsoSeq data. It was also clear that this transcript shared a transcription start site with at least one known, spliced transcript at the same locus.

6. The authors might discuss other approaches that could be used for orthogonal validation (e.g. ribosome profiling) of their results and why these have not been used. They might also point out that their approach is generally rather conservative. This is not a criticism per se but it does warrant some mention as there are sensitivity/specificity issues with both the RNA-Seq and MS approaches used.

Authors' response: We have made moderate revisions to the discussion to better highlight and explain the conservative approach taken in analysis of the RNA-Seq data and the "expressed transcriptome." Regarding MS/MS analysis, the criteria used for identification and acceptance of peptide hits from "known" proteins are relatively standard in the field, and necessary given that MS/MS is prone to false positives. Reporting of novel or non-canonical peptides calls for an even more stringent approach due to their very nature – we have revised the text to further highlight and explain that this allows a high level of confidence in the novel peptides we do report. The inclusion of the stringent criteria is further discussed in lines 195-199 and 257-272 of the revised MS.

7. There remains an overall lack of context in this study. The authors mention at least three other recent efforts to profile/annotate the MDV transcriptome but does not discuss the data presented here relative to those. While a comprehensive comparison is probably beyond the scope of this study, it would still be useful to have some discussion of similarities and differences. In studies of other alphaherpesviruses, it has generally been seen that while expression levels of viral transcripts and proteins may differ between cell types, viral strains, and experimental models, the breadth of the transcriptome (and thus the inferred proteome) remains consistent. If that is not the case for MDV then that would constitute a significant and interesting finding, particularly if it can be demonstrated that novel transcripts/proteins are expressed in live infected chickens that have never been seen in cell culture models.

Authors' response: As mentioned in a previous comment, we have done additional comparative analysis and included an additional table directly comparing our results to those of previous work on a gene-by-gene basis. We have also added discussion of the major differences in the Results/Discussion and include S10 Table for direct comparison to formers studies.

Part III – Minor Issues: Editorial and Data Presentation Modifications

Please use this section for editorial suggestions as well as relatively minor modifications of existing data that would enhance clarity.

<u>Reviewer #1:</u>

A comparative table might be useful to highlight results that are different between the current and previous work.

Authors' response: S10 Table has been added – see previous responses.

Reviewer #2:

1) The supplementary figures contain a lot of valuable data and provide a good overview of the transcriptome, peptide, splice variants aso. Unfortunately, the resolution of the supplementary figures S1-4, S6-8 and S11-20 is very low and many detail are poorly visible or blurry. As this information is important, the authors must be improved the resolution of these figures.

Author's response: Thank you for noticing this. During production of the figures, we lost resolution during conversion to Tifs. We have ensured that either the vector format or a high-resolution export of these figures has been uploaded for review as part of the revised submission. Also, we have included the whole UL region as two separate figures (Figs 2 and 3) to maintain readability.

2) When the authors describe the recombinant virus in the Material and Methods, they must include the information on the strain (RB-1B). It is nice to know the modifications made in the virus genome, but the strain is even more important.

Author's response: We apologize for excluding this information. We have not included this information on lines 791-792, as well as updated the viruses used in this study, with the inclusion of a separate animal experiment (Lines 787-792).

3) The authors state that they infected 12 chickens and kept 14 uninfected animals. Were samples pooled from several chickens to obtain the 3 and 6 replicates for MS and RNA-Seq respectively? Or were not all of these chickens used in the study? Please provide more info on this aspect.

Author's response: Only feathers from the same chicken were pooled. While confirming the details of sample collection, we realized we were incorrect in the number of birds used in the original description. For all groups, 15 chickens were used. We only selected birds showing a high level of virus replication in FFE. Not all chickens were used in the study for RNA and protein sampling. Additional information is included on lines 802-817.

4) Line 20: "little is known about the viral genes that mediate transmission, mostly due to their close relationship to their natural host." It is not clear what the author means with close relationship. Please clearly phrase this sentence.

Author's response: We have rephrased this to be clearer and now reads, "…mostly due to a lack of natural virus-host model systems." (Lines 20-21 of revised MS).

5) Line 56: the statement that "most herpesviruses are primarily cell-associated in cell culture and within the host" is not correct. Most alphaherpesviruses efficiently spread via cell free virions. Please correct…

Author's response: We understand the confusion in this statement. We have removed the "cell culture" part to be more accurate and include context in this statement. It now reads (lines 59-61 of

revised MS), "In addition, most herpesviruses are primarily cell-associated within the host where virions are delivered through cell-cell junctions and tunneling nanotubes [2], likely to evade the host immune system [3].

Reviewer #3:

Line 24: The authors should more clearly define 'fully productive replication' - I think they mean this results in virus being shed outside of the host but they language is somewhat confusing as the virus can also replicate and spread between other cells (which might be considered by some as fully productive replication).

Author's response: Thank you for this comment. With MDV, we typically consider replication in FFE cells as "fully productive" in that the "full" virus life cycle is completed, with the production of infectious, cell0-free virus. All other cells are considered "semi-productive" since no infectious cell-free virus is produced, but cell-to-cell spread is possible. To avoid this confusion, we removed "fully" to describe MDV replication in FFE cells.

Line 69: The alphaherpesvirus family is very large with only a few members well studied. The authors should clarify that 'of the well studied herpesviruses, MDV is the only one which appears strictly cell-associated'

Author's response: Thank you for noticing this. We have re-worded as suggested. We have replaced wording was modified as suggested from "most other alphaherpesvirus," to "other well-studied" in the revised MS (line 72).

Line 79: Relevant studies should be cited here.

Author's response: We have included reference 15 of the revised MS for this statement.

Table S4: The fraction column (I) shows 0 for all datasets when this is obviously not true. The authors should also include the fraction of reads aligning to the host genome and the fraction that aligns to neither host nor virus.

Authors' response: While the zero values for uninfected samples in the original table were correct given the rounding used, we changed the column format to use scientific notation to more accurately present these very small but non-zero values. When making this change, it was discovered that the viral read counts in the original table had been incorrectly tabulated from individual alignments rather than aligned read pairs, roughly double-counting most pairs. This has been corrected in the table but does not further impact any results or conclusions. We have also added columns showing fractions of host-aligned and unaligned read pairs to S4 Table.

Methods: The minimum quality score applied during TrimGalore trimming of the RNA-Seq data appears very low (8?). This could lead to a number of reads with low quality 3' ends that fail to align and a loss of data. The authors should consider higher quality values (20 and 30) to see whether this improves the fraction of reads aligning and thus the overall quality and depth of the data.

Authors' response: We partially agree with this comment, but it is not always clear that aggressive trimming is beneficial for mapping-based applications. As is the case in the current work, as mapping software such as that used (HISAT-2) can take quality scores into account and perform their own clipping as needed. There are, in fact, published results indicating the opposite, i.e., aggressive trimming can decrease quantitative accuracy of mapping-based analyses. See for example:

https://doi.org/10.1093/nargab/lqaa068

https://doi.org/10.1186/s12859-016-0956-2

Additionally, with the columns added to S4 Table as suggested above, it is more readily apparent that overall mapping percentage is quite high (93-98%) actually. We therefore respectfully disagree that any choices regarding trimming warranted a complete re-do of the RNA-Seq data processing and interpretation.
